# Supplementary material for: Insights into natural neocentromere evolution from a cattle T2T X chromosome
Source: Nat Commun. 2025 Nov 28;16:10745. doi: 10.1038/s41467-025-65778-w (PMC12663436; doi:10.1038/s41467-025-65778-w)
Supplement: Supplementary file 1 — Supplementary Information [file 41467_2025_65778_MOESM1_ESM.pdf]

Supplementary Information

Insights into natural neocentromere evolution from a cattle T2T X chromosome

*Paulene S. Pineda[1], *Callum MacPhillamy[1], *Yan Ren[1], Tong Chen[1], Luan Zhong[2], David L. Adelson[2], Carey Dessaix[2], Jose Perez-Silva[3], Leanne Haggerty[3], Fergal J. Martin[3], Cynthia D.K. Bottema[1], Wayne S. Pitchford[1], Benjamin D. Rosen[4], Timothy P.L. Smith[5], Wai Y. Low[1]

[1]Davies Livestock Research Centre, School of Animal and Veterinary Sciences, University of Adelaide, Roseworthy, SA 5371, Australia

[2]School of Biological Sciences, University of Adelaide, Adelaide, SA 5005, Australia

[3]European Molecular Biology Laboratory, European Bioinformatics Institute, Wellcome Genome Campus, Hinxton, Cambridge CB10 1SD, UK

[4]Animal Genomics and Improvement Laboratory, ARS USDA, Beltsville, Maryland, USA

[5]US Meat Animal Research Center, ARS USDA, Clay Center, Nebraska, USA

Corresponding Authors: Wai Y. Low (wai.low@adelaide.edu.au)

*These authors contributed equally to this work.

Table of Contents

Supplementary Information **0**

Supplementary Notes **6**

Supplementary Note 1. Selecting the best assembly run 6

Supplementary Note 2. Improving the genome assembly quality 6

Supplementary Note 3. Gene annotations of UOA_Wagyu_1 and its comparison with ARS-UCD2.0 and ARS-UCD1.3 6

Supplementary Note 4. rDNA copies 8

Supplementary Note 5. Telomere length variations 8

Supplementary Note 6. CENP-A enrichment in the bovine satellite repeats and X centromere 8

Supplementary Note 7. Autosomal bovine satellite repeats 9

Supplementary Note 8. Evidence for the X chromosome centromere 9

Supplementary Note 9. Finding the monomer in the X chromosome centromere and its large inverted repeats 10

Supplementary Note 10. CpG profile of X chromosome centromere 11

Supplementary Note 11. The cattle X-Y PAR and non-PAR synteny, conserved PAR genes between cattle, human and primates and conserved expression in testis 11

Supplementary Note 12. MCG results 12

Supplementary Note 13. Generating and characterising a catalogue of Wagyu SVs 12

Supplementary Note 14. SV description 13

Supplementary Methods **13**

Sequencing of the trio (Tuli x Wagyu) 13

Error correction of ONT reads 14

Genome assembly and polishing 14

Assembly evaluation 14

Finding previously unseen genes in UOA_Wagyu_1_Y 14

rDNA copies 15

CENP-A enrichment analysis 15

CpGs within centromeric vs non-centromeric regions 16

Satellite repeats in autosomal centromere 16

X chromosome centromere repeat identification 17

Methylated CpG and CpG enrichment analysis 17

Conservation of pseudoautosomal regions (PARs) 19

Testis conserved and cattle specific gene expression 19

10x coverage is insufficient to study centromere variation 19

MCG method..20
Long-read sequencing of 20 Wagyu samples...20
Detection of high-confidence SVs 20
Pangenome graph construction and SV extraction..21
Supplementary Figures 22
Supplementary Fig. 1. Telomere and X chromosome visualisation. a, A bedgraph of the telomeric repeat signals at a 10-kb window of UOA_Wagyu_1 and ARS-UCD_2.0 using tidk0.2.0. The alternating gray shades delineate the boundaries of each chromosome. **b,** Moddotplot visualization of X chromosome. Black arrow indicates an example of a low sequence similarity repeat block. The X centromere specific repeat (red box) shows alignment to this region with sequence identity >90%, confirming the location the X centromere...... 22
Supplementary Fig. 2. Centromere structure of all autosomes. Each color in the bar represents the seven satellite repeats. Chromosomes boxed in black represents structure 1 that is characterized by patterns of SATII, SATIV, SATI and interspersed SATIII and SATV. Chromosomes boxed in violet represents structure 2 that is characterized by patterns of SATII, SATIV, interspersed SATI and SATVI and tandem repeats of SATI. These are for all autosomes with complete and incomplete centromeres. Chromosomes boxed in gray remain undetermined due to the absence of SATII, which may or may not have interspersed SATI and SATVI. Their centromere structure category is also indicated. The centromere in BTA20 is not resolved. 23
Supplementary Fig. 3. Location of the X centromere specific repeats. The blue line shows the 2,898 bp (XCTR1) and 6,558 bp (XCTR2) repeats, whereas the red bars show the positions of the XCTR3 in the X chromosome... 24
Supplementary Fig. 4. Transposable elements in the X chromosome. Location of transposable elements (TEs) in the comprehensive ab initio repeat pipeline (CARP) consensus sequences of the X centromere region, analyzed under 99% sequence similarity and a minimum length of 400 bp. This figure illustrates the eight consensus sequences identified within the 12 Mb centromere region. The yellow boxes indicate the consensus family specific to the X centromere, blue boxes indicate regions covered by transposable elements based on RepBase data, with arrows representing the direction of the matched transposable element sequences. Arrows pointing to the right indicate a 5' to 3' direction, while those pointing left indicate the reverse. Three consensus sequences are chimeric, with two fully covered by TE fragments aligned in the same direction. These chimeric sequences, originating from diverse transposons, display unique structural characteristics. 25
Supplementary Fig. 5. Ratio of ratios for observed (Obs) and expected (Exp) CpGs compared to observed and expected TpGs. Scatterplot showing the ratios of (Obs_CpG/Exp_CpG) / (Obs_TpG/Exp_TpG) for each centromere. BTA20 is missing due to not being able to confidently identify the centromere. The Y-axis denotes the ratio and the X-axis denotes the chromosomes.. 26
Supplementary Fig. 6. Comparison of the Wagyu X and Y cattle chromosome pseudoautosomal regions (PARs) and adjacent sequence. The blue dots are forward alignment, and red dots are reversed alignment. The x-axis is X chromosome and y-axis is Y chromosome. The cattle PAR on X is between X: 166757707-173598413 (reversed) and Y: 20023-6819277 according to the alignment. The X and Y chromosome PAR genes (dark blue) share the same gene content and gene order. Gene families *OBP* and *BDA20* are highlighted

in green and yellow. New gene *PRP* identified on Y chromosome is highlighted in red. *SHROOM2* is located distal to the pseudoautosomal boundary (PAB) on the BTAX, which is found inverted on the Y chromosome X-d1 region. Color scale of the heatmap from blue to red indicates increasing sequence similarity. 27
Supplementary Fig. 7. The alignment plot between X and Y chromosomes. The PAR region aligns at both termini of the X and Y chromosome but at opposite ends (q-arm vs p-arm). Blue color indicates opposite orientation (-) whereas green indicates forward orientation (+). 28
Supplementary Fig. 8. Gene expression. a, Testes-expressed genes in sex chromosome for PAR regions across species. The genes are listed according to the order of cattle genes. Btau is *Bos taurus*, Hsap is *Homo sapiens*, Ptro is *Pan troglodytes*, Ggor is *Gorilla gorilla* and Ppyg is *Pongo pygmaeus*. **b,** The expression of *BDA20* (ENSBTAG00000016820) in cattle tissues grouped for mature and immature cattle. We grouped the mature cattle equal to or older than one year old. There were 3107 samples of 91 types of tissues on the boxplot. Color scale of the heatmap from red to green indicates increasing mean count on a logarithmic scale. 29
Supplementary Fig. 9. Pathway enrichment analysis. a, Pathway enrichment for genes within the X centromere. **b,** Pathway enrichment of genes that overlap with Wagyu-specific hotspots. Enriched terms identified among the 509 protein-coding genes that overlap with Wagyu-specific structural variant hotspots. Adjusted P value of Olfactory transduction = 0.04. Color scale from red to blue indicates decreasing adjusted P value. 30
Supplementary Fig. 10. Centromeric repeat clusters in autosomes. The figure illustrates the method in identifying the centromeric region (blue box) through bovine satellite repeat clusters (green box). Given the acrocentric chromosomes of the cattle, the telomere region (yellow box) is adjacent to the bovine satellite repeats (orange box) which marks the start of the centromeric region. Repeat clusters were identified as consecutive satellite repeats within 100Kb distance. The end boundary was identified as the last instance of the satellite repeat within the repeat cluster. Consecutive satellite repeats within the 100 kb but were <20Kb in total size were excluded and not classified as a repeat cluster. 31
Supplementary Fig. 11. **GenomeScope Profile**. K-mer analysis profile of the UOA_Wagyu_1 exhibiting two peaks indicating a heterozygous genome using GenomeScope2. The first peak (first dashed line) represents heterozygous with 25x coverage (single copy) and the second peak (second dashed line), represents homozygous k-mers at 50x coverage (duplicated copies). 32
Supplementary Fig. 12. **Genome quality assessment plot of the Wagyu and Tuli genome assemblies generated using Merqury**. (a) The k-mer spectra plots of the haplotype-resolved genome assemblies displaying the k-mer distribution of the F1 read set to its corresponding haplotypes. The red graph represents Tuli assembly-specific k-mers, the blue graph shows Wagyu-specific k-mers, and the green graph depicts k-mers shared between the two breeds. (b) The hapmer blob plot showing the effective phasing of the assemblies. The size of the bubble represents the size of the total k-mers. 33
Supplementary Fig. 13. Gene annotation comparison of three assemblies. Bar plot showing the different frequencies of annotations across UOA_Wagyu_1_Y (blue), ARS-UCD2.0 (orange) and ARS-UCD1.3 (green). The X-axis denotes the different annotations, and the Y-axis denotes the count. The data value is above each bar. 34

Supplementary Fig. 14. Gene annotation comparison of three assemblies focusing on the X chromosome. Bar plot showing the different frequencies of annotations across UOA_Wagyu_1_Y (blue), ARS-UCD2.0 (orange) and ARS-UCD1.3 (green). The X-axis denotes the different annotations, and the Y-axis denotes the count. The data value is above each bar. 35

Supplementary Fig. 16. Different groups of the SATI, SATII and SATVI repeat satellites based on their percent identity in our T2T chromosomes. The plot shows the different groups of satellite repeats. a, SATI into three as GroupA (≥95%), GroupB (<95% & >90%), GroupC (≤90%) and SATVI into two as GroupA (≥98%) and GroupB (<98%). The horizontal line is a guide for the 90% and 95% sequence identity. b, two group group of SATII with GroupA (≥98%) and GroupB (<98%). The different groups of the satellite repeats are associated with specific locations in the centromere, and across the genome for SATVI group B. 37

Supplementary Fig. 17. **Location of transposable elements in the CARP** consensus sequences of the X centromere region, analyzed under 99% sequence similarity and a minimum length of 100 bp. 38

Supplementary Fig. 18. Location of transposable elements in the CARP consensus sequences of the X centromere region, analyzed under 99% sequence similarity and a minimum length of 50 bp. 39

Supplementary Fig. 19. **X chromosome self-alignment plot generated using ModDotPlot**. The plot displays 14 distinct repeat blocks of varying lengths within the X chromosome region, filtered to show only sequences with greater than 90% similarity. The color gradient on the right represents sequence identity, with red indicating near-perfect identity and cooler colors reflecting lower sequence similarity. Block 2, corresponding to the centromere region of X chromosome, shows a high density of repeats, particularly short tandem repeats, as observed in the self-alignment analysis. The other labeled blocks (Blocks 1–14) indicate regions of significant repeat sequence variation across the chromosome. ... 40

Supplementary Fig. 20. Chromosome homology with ARS-UCD2.0. Dotplot alignment comparing the UOA_Wagyu_1 (y-axis) to the ARS-UCD2.0 (x-axis) for each of the chromosomes using Gepard2.1. The dotplot reveals sequence alignments and correct orientation between chromosomes, with UOA_Wagyu_1 showing additional sequences at the beginning of several of the chromosomes, corresponding to the newly resolved centromeric regions. 41

Supplementary Fig. 21. Centromeric region self-alignment dotplot. Self-alignment of the centromeric region with 5Mb upstream sequence using Moddotplot. The centromeric region were not properly assembled for chromosome 20, and hence the lack of highly identical repeat sequences (red blocks) compared to other chromosomes. Color scale from green to red indicates increasing sequence similarity. 42

Supplementary Fig. 22. Comparison of read mapping qualities (mapQ) of reads mapping to the centromere and non-centromere at three different read lengths. top left) all mapped reads available in each sample. Colours represent whether the read aligned to a centomere or non-centromere region, with symbols representing different samples. The X-axis denotes the normalised mapQ score for the region and sample and the Y-axis denotes the chromosomes. Top right) same as top left) but only considering reads ≥ 10 kb. bottom) same as top right) but only considering reads ≥ 20 kb. 43

Supplementary Table 44

Supplementary Table 1. GC content of the UOA_Wagyu_1 and ARS-UCD2.0 chromosomes and the UOA_Wagyu_1 centromeric region. 44
Supplementary Table 2. The GC content of the different bovine satellite repeats in the centromeric region 45
Supplementary Table 3. Gene annotations of the different cattle assemblies from chromosomes 1 to X and Y. Yellow highlighted rows denote rows that were common to Ensembl and NCBI annotations. These rows were used to generate Supplementary Figure 13. 46
Supplementary Table 4. Gene annotations of the different cattle assemblies in X chromosome. Yellow highlighted rows denote rows that were common to Ensembl and NCBI annotations. These rows were used to generate Supplementary Figure 14. 47
Supplementary Table 5. The coordinates and status of the 5S rDNA region found at the p-arm of the chromosomes. 48
Supplementary Table 6. The coordinates and level of the highest CENP-A peak found in the X chromosome. Normalised CENP-A level is computed by dividing CENP-A level with the size of the region. 49
Supplementary Table 7. CARP annotation of consensus sequences with 100 bp minimum length and 99% sequence identity 50
Supplementary Table 8. CARP annotation of consensus sequences with 50 bp minimum length and 99% sequence identity 51
Supplementary Table 9. Repeat blocks found chromosome X through visualization in moddotplot. An asterisk (*) indicates the centromeric region. 52

Supplementary References 53

Supplementary Notes

Supplementary Note 1. Selecting the best assembly run

Different runs of Verkko[1] and hifiasm[2] in trio mode with different combination of sequencing reads were performed. We tested different combination of input data and assembly parameters to obtain the most contiguous assemblies that have the most T2T chromosomes. The different coverages of ONT UL reads that were run with `-C0` parameter produced longer assembled telomere sequences but with slightly lower assembly quality and higher switch-error rate compared to the `-C2` parameter (Supplementary Data 1). We found the use of HERRO corrected reads[3] and the parameter `C 0` in the Verkko's consensus step enabled assembly of more T2T chromosomes. Adding HERRO corrected ONT reads as a "PacBio HiFi" input with 57x coverage and `-C0` Verkko parameter generated the greatest number of T2T chromosomes. However, when the coverage of HERRO corrected reads surpassed the HiFi reads (99x cov HERRO vs 58x cov HiFi), the assembly was poorer in quality as measured by QV, number of T2T chromosomes and number of contigs. When the HERRO corrected ONT reads were increased to 99x coverage, it also increased the genome size and number of contigs, resulting in a lower assembly quality value and a more fragmented assembly. The hifiasm assembler generated higher QV than Verkko, however, it also generated less T2T chromosomes and lower contig N50. The hifiasm run 1, equivalent to Verkko run 6 in terms of starting sequencing reads, resulted in higher QV, but also had higher switch-error rate and a larger assembly size when compared to Verkko run 6. Moreover, it generated only two T2T chromosomes compared to five T2T chromosomes in Verkko.

Supplementary Note 2. Improving the genome assembly quality

The draft genome assembly of Wagyu and Tuli were aligned with ARS-UCD2.0 reference genome[4] to identify homologous chromosomes and reorient the scaffolds. Four duplicated contigs and two mtDNA duplicated contigs were removed from the assembly. The mitochondrial genome of 16,340 bp was also assembled using MitoHiFi[5] which was 99% identical with the ARS-UCD2.0 mitochondrial genome (accession number: AY526085.1)[4].

The k-mer distribution of the Illumina short-reads revealed two distinct peaks as expected for a heterozygous diploid genome (Supplementary Fig. 11). Quality evaluation of the final genome assembly, UOA_Wagyu_1, scored a base pair QV of 54.43 and a k-mer completeness score of 99.9% using Merqury (Supplementary Fig. 12).

Supplementary Note 3. Gene annotations of UOA_Wagyu_1 and its comparison with ARS-UCD2.0 and ARS-UCD1.3

The gene sets for the Bos taurus assemblies, UOA_Wagyu_1 (GCA_040286185.1), were generated using the Ensembl Gene Annotation system (PMID: 27337980). Annotations were primarily created by aligning short- and long-read transcriptomic data to the genome. Short-read transcriptomic data from BioProjects PRJEB24380, PRJEB30263, PRJEB33849, PRJNA312901, PRJNA357463, PRJNA389288, PRJNA791623, PRJNA800088, PRJNA800822, PRJNA805180, PRJNA809209, PRJNA810710, PRJNA818914, PRJNA821268, PRJNA823050, PRJNA849569, PRJNA873056, PRJNA930909, PRJNA944650 and long-read data from BioProject PRJNA386670, PRJNA565797, PRJNA434299 were retrieved from publicly available data in the

ENA. Gaps in these annotations were filled by aligning the mammalian SwissProt proteins from UniProt (PMID: 30395287) to the genome. These proteins are reviewed and annotated based on experimental data. Additionally, human CDS regions from the GENCODE gene set (PMID: 30357393) were mapped via pairwise whole-genome alignment. At each locus, the data were consolidated, prioritising models derived from transcriptomic data to create a final set of gene models along with a non-redundant transcript set. To distinguish between true isoforms and fragments, the likelihood of each ORF was assessed against known vertebrate proteins. Low-quality transcript models, such as those with evidence of fragmented ORFs, were discarded. In loci where transcriptomic data were sparse or incomplete, homology data took precedence, favouring longer transcripts with strong intron support from short-read data. Gene models generated through this process were categorised into three main types: protein-coding, pseudogene, and long non-coding RNA (lncRNA). Models that matched known proteins and exhibited minimal structural abnormalities were classified as protein-coding. Models matching known proteins but showing structural issues—such as missing start codons, non-canonical splicing, unusually small introns (<75bp), or excessive repeat coverage—were reclassified as pseudogenes. Single-exon models with a corresponding multi-exon copy elsewhere in the genome were identified as processed (retrotransposed) pseudogenes. If a model did not meet the criteria for the above categories, did not overlap with a protein-coding gene, and had been derived from transcriptomic data, it was classified as a potential lncRNA. Single-exon loci were excluded from the lncRNA category due to the unreliability of such models. Putative miRNAs were predicted by performing a BLAST search of miRBase (PMID: 30423142) against the genome, with the results subsequently processed using RNAfold (PMID: 18243585). Other small non-coding RNA loci were identified by scanning the genome with Rfam (PMID: 29112718), as detailed in PMID: 27337980, with results passed to Infernal (PMID: 24008419).

We compared the Ensembl gene annotation of UOA_Wagyu_1_Y with that of ARS-UCD2.0 (NCBI) and ARS-UCD1.3 (Ensembl). Looking first at the protein-coding genes, UOA_Wagyu_1_Y had 20,549 protein-coding genes annotated, whereas ARS-UCD2.0 had 21,423 and ARS-UCD1.3 had 23,740 (Supplementary Fig. 13-14; Supplementary Table 3-4). UOA_Wagyu_1_Y had more protein-coding genes annotated to chromosome 10 than ARS-UCD2.0, and more genes annotated to the X chromosome than ARS-UCD1.3 and ARS-UCD2.0. UOA_Wagyu_1_Y had the least annotated pseudogenes of the three references, 365 compared to 4292 (ARS-UCD2.0) and 526 (ARS-UCD1.3). UOA_Wagyu_1_Y tended to have the most annotations for different RNA species of the three assemblies. UOA_Wagyu_1_Y (13,517) had more than double the number of lncRNA annotations of ARS-UCD2.0 (5,551) and substantially more than ARS-UCD1.3 (8,448). As expected, given the completeness of UOA_Wagyu_1_Y, there were substantially more rRNA annotations in UOA_Wagyu_1_Y (1,398) compared to 14 and 63 in ARS-UCD2.0 and ARS-UCD1.3, respectively. Similarly, UOA_Wagyu_1_Y had the greatest number of snoRNA annotations (805) compared to 635 (ARS-UCD2.0) and 736 (ARS-UCD1.3). UOA_Wagyu_1_Y also had the greatest number of snRNA annotations, with 1,254 compared to 965 (ARS-UCD2.0) and 1,214 (ARS-UCD1.3) The 906 annotated miRNAs in UOA_Wagyu_1_Y were relatively similar to ARS-UCD2.0 (1,029) and ARS-UCD1.3 (1,007), as were the miscellaneous RNAs; 24 in UOA_Wagyu_1_Y compared to 42 and 25 in ARS-UCD2.0 and ARS-UCD1.3, respectively. Both UOA_Wagyu_1_Y (9) and ARS-UCD1.3 (9) have substantially fewer tRNA annotations than ARS-UCD2.0 (2,279). Other annotations are reported within each assembly's respective annotation file, but we have only compared those that exist in all three (Supplementary Table 3).

Supplementary Note 4. rDNA copies

The rDNA unit were comprised of 18S, internal transcribed spacer 1 (ITS1), 5.8S, internal transcribed spacer 2 (ITS2), 28S, external transcribed spacer 2 (ETS2), intergenic spacer (IGS) and external transcribed spacer 1 (ETS1) spanning a ~35 kb tandem. The consensus sequence of cattle rDNA based on 131 rDNA units detected in UOA_Wagyu_1 was 34,697 bp long. The region that spanned 18S, 5.8S and 28S showed ~99% sequence identity among the rDNA units. As expected, the IGS region varied substantially across copies (Supplementary Fig. 15). The longest tandem repeats of rDNA were 9 units that spanned 300 kb in the unplaced region. The rDNA units found in unplaced scaffolds were also found in the string graphs connecting to BTA3, BTA4, BTA11 and BTA25 (Figure 1f). This was done by identifying the nodes corresponding to the scaffolds. Although BTA4 has a telomere on the p-arm and without gaps, the presence of an rDNA at the q-arm and unplaced rDNA-containing nodes in the string graph suggest that the rDNA prevented the assembly of a T2T chromosome.

The combination of the high coverage (>58x) and high-quality PacBio HiFi reads and ultra-long Nanopore reads resolved large repeat blocks after the centromeric regions which were not previously observed in the ARS-UCD2.0. These repeat blocks were composed of tandemly repeated 5S rDNA regions in BTA14, BTA22, BTA27 and BTA28. However, BTA22 and BTA28 were not fully resolved and contain gaps within them (Supplementary Table 5).

Supplementary Note 5. Telomere length variations

There were 11,624 telomeric units assembled at the p arms and 53,341 telomeric units were found at the q arms. The average telomere length differed between the p and q arms of the T2T and TgapT chromosomes, with the q arm having a longer telomere repeat region (14 kb vs. 11 kb). The telomere length for each chromosome varied substantially too, with BTA7 having the longest telomere length (21 kb) and BTA16 (5.6 kb) having the shortest telomere length (Supplementary Table 5). The differences in the telomere length were more likely dependent on the reads obtained rather than a full representation of its lengths[6].

Supplementary Note 6. CENP-A enrichment in the bovine satellite repeats and X centromere

CENP-A is a protein that plays a role in centromere function by marking the centromeric region and signalling the kinetochore to form which ensures attachment of the spindle fiber and maintains the chromosome alignment during cell division. We used two CENP-A signal indicators which was the normalised total signal and the maximum peak of the CENP-A per satellite repeats. The SATIII contains six times higher total CENP-A levels compared to the total of the rest of the bovine satellite repeats. For X chromosome, the centromere did not show higher enrichment of the CENP-A, but six distinct peaks were found outside of the centromere boundary. We found very distinctive and high peaks of CENP-A in the X chromosome which are outside of the centromeric region. Four of these highest CENP-A peaks were also the regions with the largest CENP-A-enriched areas (Supplementary Table 6).

Supplementary Note 7. Autosomal bovine satellite repeats

The Wagyu genome assembly showed a higher GC content at 44%, compared to 42% in the ARS-UCD2.0 genome. This was due to the resolved centromeric region of the Wagyu genome assembly, which had a GC content of 54%. The most abundant repeats, SATI and SATII had an average GC content of 59% and 56% (Supplementary Table 1-2), respectively, which contributed to the high GC of the centromeric region. The SATVII has the highest GC content with an average of 60% that was adjacent to the telomeric region.

The cattle centromere is composed of tandemly repeated satellite repeats. The centromeric regions of the T2T chromosomes BTA9, BTA10, BTA21 and BTA23 spanned 24 Mb, 15 Mb, 17 Mb, and 14 Mb. Additionally, BTA4 managed to assemble through the centromere which spanned 17 Mb. About 43% and 32% of the centromeric region consisted of SATI and SATIII, respectively. Six of the bovine satellites were known but we modified the consensus repeat sequences of one satellite (SATIII) based on the repeats detected in our Wagyu reference. The SATIII and SATV were composed of the same 23 bp monomer with ~65% sequence identity. SATVII is a novel 93 bp satellite with 3-copies of the telomere unit in tandem repeat at the middle of its consensus sequence.

The bovine satellite repeats showed different clustering or groups based on their sequence identity to the consensus sequence (Supplementary Data 7). The following discussion will focus exclusively on the T2T chromosomes. The SATI was further divided into 3 groups which we named Group A, B and C (Supplementary Fig. 16). Group A consisted of repeats with ≥94% sequence identity and tandemly repeated, Group B with <95% but ≥ 90% sequence identity that were interspersed with SATVI and Group C with <90% sequence identity that were found at the end of the centromeric region. The SATI group A consisted of a total of 22,434 copies, that spanned an average of 8 Mb per chromosomes, while the group B consisted of a total of 2,453 copies found only in BTA9 and BTA23 and were interspersed with SATVI. The SATI group C consisted of 339 copies and were located at the end of the centromere. These classifications of the satellite repeats were also observed in SATVI wherein highly similar repeats with ≥98% sequence identity was interspersed with SATI (1,223 copies) and were found only in BTA23 (Supplementary Fig. 16a). While 90 copies of the SATVI <98% sequence identity in single copies were found across the genome. SATII showed ≥95% sequence identity which were tandemly repeated with a total of 3,923 copies found in all T2T chromosomes. A lower sequence identity of the SATII was found in the flanking region of the tandem arrays of SATII (<95%) comprising of 9 copies for each T2T chromosomes (Supplementary Fig. 16b). The SATIII has lower sequence identity (<90%) than the other satellite repeats, consisting of 8,361 copies, with average length of 5 Mb (Figure 2e). There were 641 copies of SATV, with BTA23 having only one copy and is only 80% similar with the consensus. The new satellite repeat that we have identified, SATVII, was found in between the telomere region and SATII, which is sandwiched within TEs and has a total of 76 copies.

Supplementary Note 8. Evidence for the X chromosome centromere

The X centromere is unveiled in full sequence for the first time at coordinates between 38 and 50 Mb on the p-arm. One evidence is the fact that the cattle X chromosome is known to be submetacentric with the X-PAR on the q-arm[7]. Of the 14 distinct repeat blocks detected on the X

chromosome, with sizes ranging from 0.87 Mb to 12 Mb, only six could be candidate X centromere, including the one situated between 38 and 50 Mb. Second, a laser microdissection of cattle X centromeres to obtain bovine X-centromere DNA was followed by fluorescence in situ hybridization experiment (FISH) using the dissected probe to confirm its location in the X centromere. Subsequent sequencing of the probe revealed a X chromosome specific repeat (AJ884576)[8]. We confirmed that this repeat is only located in the X centromere at our indicated position. Third, the results of a cytogenetic study that mapped genes located on the X-chromosome p- and q-arms is in agreement with our X centromere position[9].

Supplementary Note 9. Finding the monomer in the X chromosome centromere and its large inverted repeats

To further explore the X centromere region, we conducted a focused CARP analysis with parameters specifically optimized for it. This approach provided more detailed insights into the repeat structures within the centromere and helped identify potential patterns. Supplementary Fig. 4 presents the eight repeat consensus sequences detected by CARP, each with 99% sequence similarity and a minimum sequence length of 400 bp. While all these sequences contain fragments of transposable elements, none are fully annotated as such. The centromere is predominantly covered by Family 2 (C2), Family 6 (C6), and Family 7 (C7). The repeat Family 0 (C0), which is a specific repeat sequence within the centromere, is located at the beginning of this region. Another specific sequence, Family 3 (C3), exhibits a regular repeat pattern in the self-aligned plot, where the consensus sequence repeats in pairs, with one copy in the forward direction (inverted repeat).

As the minimum sequence length is reduced, more repeat families are detected. With a 100 bp minimum length, 13 repeat families were identified, 6 of which have over 100 repeats in the centromere region (Supplementary Table 7; Supplementary Fig. 17). When the threshold is further lowered to 50 bp, CARP identified 23 repeat consensus sequences (Supplementary Table 8; Supplementary Fig. 18), and 16 consensuses have over 100 repeats in the centromere region.

CARP analysis identified 2,190 repeat consensus sequences in the X chromosome. Of these, 80% (1,752) were fully annotated as TEs, while 4% (93) were classified as chimeric sequences. Additionally, 13% (303) were partially annotated as TEs, and two were classified as retrovirus-like elements. Finally, 1% (40) of the consensus sequences remained unclassified. In total, 64% of the chromosome are composed of TEs.

The self-aligned dotplot of the X chromosome revealed 14 distinct repeat blocks with high sequence identity (>90%) with size ranging from 0.87 Mb to 12 Mb dispersed across X chromosome (Supplementary Fig. 19; Supplementary Table 9). The largest repeat block, Block 2, corresponded to the centromeric region of X chromosome, located between the 38 Mb and 50 Mb. These repeat blocks were abundant in various repeats and densely populated with TEs. Based on CARP results, a significant portion of this region is composed of TEs, with ~8.6Mb or 72.15%.

A total of 4,527 repeats were identified by TRF, consisting of short sequences with low copy numbers and a wide variety of repeat types. About 21 of these tandem repeats contains >100 copies, however, with low sequence identity from 61-85% identity.

A total of 6,560 inverted repeat pairs were identified, in which the left arm has more than or equal 95% sequence identity to its right arm pair (Figure 3d) The size of inverted repeats ranged from 10 bp to 620 kb. We did not find the bovine satellite repeats that were hallmarks of autosomal centromeres in the X centromere.

The largest inverted repeat in the X centromere was at X:41666725-42324966, which was 658 kb long (left arm) and had 98% sequence identity between arms (Figure 3d). We aligned the rest of the inverted repeats to this largest repeat and around 73% (4,270) of all repeats identified on X centromere aligned to it with 95% sequence identity, with at least 80% of the query alignment coverage length. In addition, three highly similar (99%) large inverted repeats of ~620 kb (X: 39725293-40344528, X: 43080361-43696976, X: 40753657-41369420) were highly identical, pairing with the same right arm (X: 47990311-48609658).

We also found two sequences that specifically appeared in the X centromere using CARP (Supplementary Fig. 4). A 550 bp sequence, identified as family 0 consensus, was discovered in the beginning of the X centromere using CARP. This sequence appeared 23 times in the X centromere and was unique to this region within the Wagyu genome. The 550 bp sequence was predominantly located from 38,142,077- 38,155,012 of X chromosome and is not part of the inverted repeats. Another unique sequence in the X centromere (family 3 consensus) was a 636 bp repeat, which spread across the X centromere with 35 copies. We named these repeats as XCTR4 (550 bp) and XCTR5 (636 bp).

Supplementary Note 10. CpG profile of X chromosome centromere

It has been only recently, with the completion of T2T genomes, that we have been able to analyse fully the epigenetic profile and sequence composition of centromeres. Three different window and step sizes (100 kb with 50 kb step, 1 Mb with 500 kb step, and 5 Mb with 2.5 Mb step) were tested to analyse CpG profile of the centromeric and non-centromeric regions (Supplementary Table 8). Considering all centromeres against all non-centromeric regions genome-wide, we consistently observed the centromeric regions as having a significantly lower median methylation value than the non-centromeric regions across the three window sizes tested, regardless of the sequencing technology used. Among the chromosome-wide comparisons, PacBio and ONT agreed that the centromere was significantly less methylated than the non-centromeric region of the chromosome.

There was a lack of significance at BTA2, BTA23, BTA25 and BTA29, for either ONT (BTA2) or PacBio (BTA23, BTA25, BTA29). However, the pattern of lower methylation in the centromere was maintained. The X centromere methylation signal is different to what we observed in human, where there was a lack of discernible pattern between centromere and non-centromere methylation levels.

Supplementary Note 11. The cattle X-Y PAR and non-PAR synteny, conserved PAR genes between cattle, human and primates and conserved expression in testis

The ~6.8 Mb PAR had no contig gap and was 99% identical between the X and Y chromosomes, similar to previous finding[10]. Apart from a single gene, basic proline-rich protein (*PRP*), which was unique in the Y PAR, the X and Y chromosomes shared 31 genes in the same order. These 31 common genes were comprised of two multi-copy gene families, odorant-binding protein (OBP)

with four copies and bovine dander allergen (BDA20) with three copies, and 29 single-copy genes (Supplementary Table 11, Supplementary Table 12).

Four genes, HSFY, TSPY, ZNF280BY, PRAMEY had 40, 102, 22 and 33 copies, respectively in the ampliconic regions. As expected, none of these four ampliconic genes were found on the X chromosome. However, two genes, PRSS52P and OR4C6Y, were labelled on the X-degenerate regions, but had no homologs on the X chromosome. The gene, RBMY, was in the ampliconic region but it also has three X-Y homologs (RBMX, and two copies of RBMX2).

Chimpanzees, gorillas, and bonobos have a common gene (SPANX), with two, seven, and 24 copies on X chromosome, respectively. The 30 conserved genes among the eight species share the same order.

Among the 17,007 genes identified in cattle, 832 were expressed on the X chromosome, while 149 were expressed on the Y chromosome. Twenty-eight of them were expressed in the X-PAR and 26 of them expressed in the Y-PAR. There were 16,410, 17,234, 17,162 and 16,843 genes expressed in the testis of human, chimpanzee, gorilla and Bornean orangutan, respectively (Supplementary Table 15). Eighteen conserved genes were expressed in testis in all five species on the X-PAR (Figure 5b).

For cattle X-Y homologs on PAR, 24 were expressed in testis on both X and Y chromosomes. OBP, ASMT and ZBED1 were only expressed on the X-PAR whereas DHRSX and PRB were only expressed on the Y-PAR. For cattle X-Y homologs on non-PAR, EIF1A, USP9, ZRSR2, UT, DDX3, SHROOM2, ZF, EIF2S3, TXLNG and RBM were expressed in testis on both X and Y chromosomes. OFD1 was only expressed on the X-non-PAR whereas no X-Y homolog gene was only expressed on the Y-non-PAR.

Supplementary Note 12. MCG results

To determine whether the MCG method indeed captured more genetically diverse 20 animals within the Wagyu population, we compared the number of unique variants, i.e. non-reference alleles, observed from Versa50K genotype data between the 20 animals identified by MCG and batches of 20 randomly selected animals over 50 iterations. We consistently observed more unique variants among the 20 MCG-selected animals compared to the randomly chosen animals, suggesting that the MCG method is a suitable means to determine the most diverse animals when a limited number must be chosen (Figure 6a). We used the MCG method to select 20 animals that best captured the genetic variation within the wagyu population as we have demonstrated it consistently outperforms random sampling.

Supplementary Note 13. Generating and characterising a catalogue of Wagyu SVs

Using UOA_Wagyu_1_Y as the backbone to the graph made up of 13 assemblies, we identified 283,348 PAVs ("graph PAVs"), each more than 50 bp. We also identified 5,840 PAVs ("WAG assembly PAVs") unique to UOA_Wagyu_1_Y. We used these graph and WAG assembly PAVs to identify SV hotspots throughout UOA_Wagyu_1_Y. This analysis was repeated using our high-confidence SV set from the 20 Wagyu ONT data. We identified 228 hotspots from the "graph PAVs" ("graph hotspots"), 97 from the "WAG assembly PAVs" ("WAG assembly hotspots") and

161 from our high-confidence SV set (“population hotspots”). Following this, we merged the 97 “WAG assembly hotspots” with the 161 “population hotspots” and selected all hotspots that did not overlap with any of the "graph hotspots” by greater than 50% of their length.

Given the challenge in resolving these centromeric regions, it is unsurprising that we found 10x coverage from ONT reads insufficient to study centromere variation in our 20 Wagyu samples.

Supplementary Note 14. SV description

We examined the composition of the 49,610 SVs identified on UOA_Wagyu_1_Y and the 47,213 SVs identified on ARS-UCD2.0. We found that regardless of reference genome used, most SVs were insertions (22,607 from UOA_Wagyu_1_Y; 23,376 from ARS-UCD2.0) and deletions (26,687 from UOA_Wagyu_1_Y; 23,310 from ARS-UCD2.0) (Supplementary Table 24). Inversions were the next most frequently observed SVs (175, 294), followed by duplications (105, 160) and lastly breakends (36, 73).

We further categorised these SVs based on whether they were found in all samples, most samples (greater than ten but less than 20), some samples (greater than two but less than ten) and few (only two samples) (Figure 6d). Focusing on UOA_Wagyu_1_Y, among insertions, 19,128 SVs were found in three to twenty samples with 3,479 being unique to a pair of samples. This trend was observed in the remaining SV types, with the majority of deletions (22,072) occurring within three to twenty samples; 4,615 were unique to a pair of samples. Among the duplications, 38 of 105 were unique to a pair of samples, similarly 44 of 175 inversions were found only in pairs of samples and finally, 11 of 36 breakends were unique to a pair of samples (Supplementary Table 24).

Supplementary Methods

Sequencing of the trio (Tuli x Wagyu)

A Wagyu dam was inseminated with semen from a Tuli bull. This is to produce a hybrid calf for the trio-binning method using a diverse lineage and generate a haplotype-resolved assembly of the Tuli and Wagyu genomes. The Tuli cattle, an African breed, is renowned for its adaptability, while the Australian Wagyu cattle is prized for its high marbling and market value. This genetic diversity enhances the effectiveness of the trio-binning method in genome assembly while providing two genomes from each breed. A male Tuli x Wagyu F1 hybrid calf was born, and blood was extracted from it when it was five months old. Blood was also extracted from the dam. For the F1 progeny, DNA was extracted from the blood using the Circulomics Blood UHMW protocol for HiFi sequencing and from the Monarch® HMW DNA Extraction Kit for Tissue for Oxford Nanopore Technologies (ONT) sequencing. For HiFi sequencing, the DNA was sheared with Megarupter at speed 17 and then size selected on the Sage Science Blue Pippin to remove all fragments below 15 kb. The whole genome and metagenome libraries of SMRTbell prep kit 3.0 was used. For ONT, the LSK110, LSK114, NBD114 and ULK114 sequencing kits were used. The Phase Genomics ProximoTM Hi-C (Animal) Kit was used for Hi-C sequencing library preparation. For the dam, DNA from the blood was extracted using an in house salting out method. For the sire, DNA from the semen was extracted using Phenol/Chloroform method according to the protocol recommended by PacBio (pacb.com/wp-content/uploads/Preparing-Genomic-DNA-from-Sperm.pdf).

Error correction of ONT reads

About 57x R10.4.1 ONT reads were filtered for minimum read length of 10 kb and minimum average read quality of QV10. These filtered reads were selected for HERRO error correction using the model called model_v0.1.pt. A further ~99x R10.4.1 ONT reads were filtered for minimum read length of 30 kb and minimum average read quality of QV10, and these reads were also selected for HERRO error correction using the same model. These error corrected reads were used as HiFi equivalent reads when running Verkko[1] and hifiasm[2].

Genome assembly and polishing

Eight different runs of Verkko (v2.0) were performed with different coverages of sequencing reads and assembly options (Supplementary Table 1). The chosen run was run 5, which assembled the greatest number of T2T chromosomes. The draft assembly was then aligned to ARS-UCD2.0[4] using minimap2 (v2.26)[11] to confirm chromosome homology and orientation. In instances where a UOA_Wagyu_1 chromosome was reverse oriented compared to ARS-UCD2.0, CombineFasta[12] was used to reorient it to follow ARS-UCD2.0 and conform with the established cytogenetics for cattle. Additionally, the assembly was aligned to ARS-UCD2.0 with Gepard (v2.1)[13] to produce dot plots that allowed visual inspection of mis-assemblies (Supplementary Fig. 20). For polishing the assembly, PacBio HiFi reads were aligned to each draft assembly of Tuli and Wagyu using minimap2 with the parameters `-a -asm20`. The output BAM files were sorted and indexed using SAMtools (v1.17)[14]. The SNP and indel variants were called with DeepVariant (v1.6.1)[15].

Assembly evaluation

The assemblies were evaluated based on T2T chromosomes, telomeres, N50, Merqury QV and switch-error. The telemore statistics were estimated using the Vertebrates Genomes Project's (VGP) script 'telomere_analysis.sh' with the parameters "0.5 5000"[16]. The telomere repeat counts were determined by counting the canonical vertebrate telomere (TTAGGG) on either strand within the putative telomeric region. N50 and genome size were calculated using QUAST (v4.5)[17]. Merqury (v1.3)[18] was used for QV and switch-error assessment. Assembly gaps were identified with seqtk (v1.3)[19].

Finding previously unseen genes in UOA_Wagyu_1_Y

The gene annotation for ARS-UCD2.0 was split into per chromosome GTF files and ARS-UCD2.0.fa was split into per chromosome fasta files. UOA_Wagyu_1_Y was equally split into per chromosome fasta files. Liftoff was then used to align each ARS_UCD2.0 chromosome and gene annotation to the corresponding UOA_Wagyu_1_Y chromosome. An example of the command is:

```
liftoff –g $ARS.UCD2.0.chr1.gtf $UOA_Wagyu_1_Y.chr1.fa $ARS-
UCD2.0.chr1.fa
```

rDNA copies

Searches for rDNA by using the keyword "rDNA cattle" in the NCBI database, and then filtering the results for "DNA" and "Bos taurus" retrieved three sequences: MT362686.1, MT362686.1 and DQ222453.1[20]. Only DQ222453.1 contained the 18S, 5.8S and 28S, and hence these were selected to identify rDNA sequences in the UOA_Wagyu_1 genome. A complete copy of the rDNA is defined as a region that has 18S, 5.8S and 28S in the right order with minimum 85% percent identity, 80% query coverage and e-value of 1e-10. The ITS1 and ITS2 of the DQ222453.1 did not align well with our assembly, and hence we have not added these regions to identify the completeness of rDNA in the UOA_Wagyu_1. We observed that the cattle rDNA unit was ~35 kb, which represented the complete region of the rDNA, including the external transcribed spacer (ETS) and intergenic spacer (IGS) regions[21]. We selected one representative region of the ~35 kb rDNA on BTA11, aligned it with BLASTn to the UOA_Wagyu_1 genome assembly. We extracted each region that represented a rDNA unit (~35 kb) that aligned between consecutive 18S sequences. If the contig contains a single copy and the rDNA unit is shorter than 30 kb, these were filtered out. These regions were extracted using SAMtools (v1.17) and were reverse complemented if in "-" strand using `seqkit -p -r -t dna`[22]. After extracting all the rDNA sequences >30 kb, these sequences were aligned using `mafft –auto`[23]. The consensus sequence was generated by using ` hmmemit -c` after building a hmmr profile by running `hmmbuild –dna`[24] to the mafft alignment file.

As the ETS and IGS of cattle has not been characterized yet, these regions were determined in the cattle consensus sequence using the human rDNA (Genbank: U13369.1) as reference. To identify the ETS region in the cattle consensus rDNA sequence, alignment was done using mafft with parameters "--globalpair --maxiterate 1000". The IGS region was determined to be the region in between the 3'ETS and the 5'ETS. The unplaced scaffolds containing rDNA were also determined in the assembly string graph by visualizing in Bandage[25] identifying its corresponding nodes.

CENP-A enrichment analysis

We obtained CENP-A ChIP-seq data from Olagunju et al. (2024)[26]. The filtered reads were aligned to the reference genome using Bowtie2 (v2.5.1)[27] with the command, `bowtie2 -k 100 -x $reference_genome -1 $filtered_reads_1.fastq.gz $filtered_reads_2.fastq.gz`. Alignments were then sorted with SAMtools (v1.19) and deduplicated with Picard MarkDuplicates (vX.X) (https://broadinstitute.github.io/picard/). We then generated input bedGraph files for SEACR[28] following the authors' recommended method. The SEACR peak caller was then used to call CENP-A peaks using stringent and 10% filter options, `bash SEACR_1.3.sh $target.bedGraph 0.1 non stringent $output.bed`.

Two different types of CENP-A level were analysed in the study: the total signal and the maximum signal called by (Sparse Enrichment Analysis for CUT&RUN) SEACR. The output data of SEACR contains 6 columns which are the following: 1) chromosome, 2) start coordinate, 3) end coordinate, 4) total signal contained within the denoted coordinates, 5) maximum bedgraph signal attained at any base pair within the denoted coordinates, 6) coordinates represented by the maximum bedgraph signal. The total signal and max signal were retrieved in the SEACR output, which is the fourth and fifth columns, respectively. The total signal is contained within a coordinate, which were the CENP-A enriched regions. The CENP-A signal per bases were

computed by dividing the total signal CENP-A level to the enriched region length. Since the enrichment level is denoted by coordinates, we intersect the regions to the satellite repeats coordinates. Intersections that cover <50% of the satellite repeat size were filtered out. The normalised CENP-A level was then taken as the CENP-A per bases. For the CENP-A max signals, the coordinates were intersected with the satellite repeats and the values were as is.

CpGs within centromeric vs non-centromeric regions

We tested whether CpGs were enriched within centromeric compared to non-centromeric regions. To do this, we generated a centromeric region fasta file and a non-centromeric region fasta file. This was achieved by generating a BED file of the centromeric boundaries defined above. We then took the complement of these regions from UOA_Wagyu_1_Y using BEDtools complement to capture the non-centromeric regions. We then extracted the fasta sequences for these regions with BEDtools getfasta. Next, we used Jellyfish (v2.3.1)[29] to count kmers. An example of the command used is below:

```
jellyfish count -m 2 -s 16 -o UOA_Wagyu.centromeres.2mer.jf -t 4
UOA_Wagyu.centromeres.fa | jellyfish dump -c -t
UOA_Wagyu.centromeres.2mer.jf > UOA_Wagyu.centromeres.2mer.tsv
```

To test if the frequency of CpG dinucleotides was significantly different in centromeres compared to non-centromeres, we constructed a 2x2 contingency table of the form:

	**Is_Centromere**	**Not_Centromere**
**Is_CpG**	Is_Centromere_Is_CpG	Not_Centromere_Is_CpG
**Not_CpG**	Is_Centromere_Not_CpG	Not_Centromere_Not_CpG

Finally, we performed a linear regression to determine if the expected CpG count was correlated with the observed CpG count. The observed CpG count was given by the number of occurrences of ‘CG’. The expected CpG count was calculated by:

$$CpG_{expected} = \frac{Ccount \times Gcount}{len(region)}$$

Satellite repeats in autosomal centromere

We run RepeatMasker to the UOA_Wagyu_1 assembly to determine the bovine satellites annotated by the RepeatMasker[30]. Known satellite repeats from the literature (Supplementary Table 28) were then aligned to the Wagyu genome assembly using BLASTn with the parameters “-evalue 1e-10 -perc_identity 85”. We generated a gff file for both BLASTn and RepeatMasker results and visualised the annotations using IGV (v2.17.4)[31]. There were six known bovine satellites. We identified that the SATI or Sat1.715 was equivalent to BTSAT4. BTSAT5 was a subsequence of BTSAT4. SATII or Sat1.723 was equivalent to BTSAT6 in RepeatMasker. SATIII or Sat1.706 comprises of the SAT1.720, BTSAT1, BTSAT2 and BTSAT3. SATIV was Sat1.709. SATV or Sat1.711a comprised of BTSAT2b and BTSAT3b. SATVI was 1.711b.

We also observed that the SATIII contained known repeats that were fragmented, hence could still be extended. We counted the repeat sequences' kmers from the contigs using kmc[32] with parameters, "-k151 -ci20 -cs5000" in the SATIII-SATV regions and then ran Satellite Repeat Finder (SRF commit version: e54ca8c)[33] to get the consensus sequence and identified the satellite repeat monomer. We updated the SATIII to be a 2,387-bp repeat monomer. Manual inspection of the sequences from the telomere of the p arm to the SATII of centromeric region revealed a repeating pattern that was not one of the known six satellites. We ran Tandem Repeat Finder (TRF)[34] with parameters "2 7 7 80 5 200 2000" for the region between the telomere and SATII. We found a novel repeat, which we called SATVII, and we built its consensus by using the same method as above using kmc and srf. The kmc parameters were "-k90 -ci5 -cs500" since these are smaller repeats. This resulted in a 93-bp repeat satellite monomer.

The custom library to identify the repeats in the UOA_Wagyu_1 was created by extracting fasta sequences specific to the Bos taurus lineage from the combined default RepeatMasker library and RepBase RepeatMasker Edition version 20191026 by using "famdb.py" in the RepeatMasker utils. The satellite repeats were also updated with new and extended sequences and simplified naming convention (SATI, SATII, SATIII, SATIV, SATV, SATVI and SATVII). After establishing the satellite repeats, a custom library was created and used to identify repeats using RepeatMasker with the parameter "--lib". The centromere boundaries were again updated using the custom library. The overlaps between repeats were removed by using "RM2Bed.py" in the RepeatMasker utils with parameters "--ovlp_resolution 'higher_score' --max_divergence 40", which generates a bed file of the repeat coordinates.

Additional evidence for the repeat clusters in the autosomal centromeres were obtained using ModDotPlot (v0.8.2)[35] with an 85% identity threshold (Supplementary Fig. 21). Since we found complex patches of repeats in the SATIII region (not tandemly repeated with the known repeats), we used SRF to determine the longest consensus sequence of the SATIII. These curated bovine satellite repeats were then added to the RepeatMasker library for searching the UOA_Wagyu_1. After determining all the repeat satellites in the bovine centromeric region, we again used RepeatMasker to align the repeats to the Wagyu genome assembly to annotate the repeats in the autosomes and determine the centromere architecture.

X chromosome centromere repeat identification

To validate that the ~12Mb repeat block visualised in ModDotPlot[35] is the X centromere (X:38,000,000-50,000,000), bovine X chromosome centromeric specific repeat (Genbank: AJ884576) were aligned to the UOA_Wagyu_1 using BLASTn with at least 90% sequence identity, 80% query coverage and e-value of 1e-10. The X chromosome centromeric repeat indeed aligned only to the X chromosome specifically from 39 Mb to 49Mb. This validated the repeat block to be the X centromere. We also run TRF to identify the monomer in X centromere however, we did not find tandemly repeated sequences like the autosome's centromeric region.

Methylated CpG and CpG enrichment analysis

The resulting BAM file was sorted with SAMtools (v1.19) and then used as input to pb-CpG-tools where we used the default parameters to identify methylated CpGs:

```
pb-CpG-tools/bin/aligned_bam_to_cpg_scores --bam
$PacBio_data.sorted.bam --output-prefix PacBio_CpG --model
pileup_calling_model.v1.tflite
```

To determine the CpG methylation levels of the ONT reads, we adopted a similar approach, first mapping the ONT reads generated for the assembly to UOA_Wagyu_1_Y using dorado (v0.6.0). We then used modkit pileup (v0.2.8) to quantify the genome-wide CpG methylation. The modkit command was:

```
modkit pileup --log-filepath UOA_Wagyu_1_Y.pileup.log \
  --threads 16 \
  --ref UOA_Wagyu_1_Y.fa \
  --preset traditional \
  $bam \
  $UOA_Wagyu_1_Y.bedMethyl
```

A series of sliding windows with window sizes of 5 Mb, 1 Mb and 100 kb, and step sizes of 2.5 Mb, 500 kb and 50 kb, respectively, were performed to extract the median methylation of each window from UOA_Wagyu_1_Y. We further classified these windows as being 'centromeric' or 'non-centromeric', with the centromere boundaries being rounded down to the nearest 1 Mb
To determine CpG methylation levels of the PacBio reads, we followed the recommendations of pb-CpG-tools. We first aligned PacBio data with the following:

```
pbmm2 align UOA_Wagyu_1.mmi $PacBio_data.bam
```

To investigate centromeric methylation differences, we rounded the centromere boundaries down to the nearest 1Mb. For example, if the end position of the centromere was 21,994,403, we rounded it down to 21,000,000 and took this as the end position of the centromere. Using these new centromere boundaries, we then binned the genome with BEDtools makewindows[36] (v2.31) with '-w 5000000' and '-s 2500000'. We then pooled methylation values within these windows to generate mean and median methylation values for each 5 Mb window. To determine with there was a significant difference in methylation level between centromeres and non-centromeres we used a Kolmogorov-Smirnov test.

Human methylation data was downloaded from https://s3-us-west-2.amazonaws.com/human-pangenomics/index.html?prefix=T2T/CHM13/assemblies/annotation/regulation/. We converted chm13v2.0_hg002_5mC_hifi_revio_modkit0.1.11.bw and chm13v2.0_CHM13_CpG_ont_guppy3.6.0_nanopolish0.13.2.bw to BED files with bigWigToBedGraph prior to analysing median methylation. chm13v2.0_hg002_CpG_ont_guppy6.1.2.bed was used as is. Human centromere coordinates were downloaded from the UCSC Genome Browser CenSat Annotation track for genome CHM13v2.0/hs1.

Conservation of pseudoautosomal regions (PARs)

To identify the PAR regions, pairwise alignments of X and Y chromosomes were carried out for cattle. Briefly, the process involved alignments with Lastz v1.04[37] with "--notransition --step=20 --nogapped --format=maf" and then visualization of the alignments with dotplot. Since cattle PARs are known but contained contig gaps[10], we used these PARs as template for BLASTn from BLAST+ (v2.14.1) to extract homologous PAR regions in Wagyu. The last-dotplot from last[38] (v1.0) package was used to plot the maf file produced from Lastz focusing on the homologous PAR regions to identify PAR boundaries.

To identify homologous genes on cattle PAR, BLASTp was used. In the case of uncharacterized genes (e.g. prefixed with LOC), their longest protein sequences were filtered to check if they were longer than 100 amino acids. BLASTp was performed for these proteins against NCBI (non-redundant protein sequences) to check for any known homologous genes. For genes with query coverage > 80% and percentage of identity > 80% in the BLASTp result, gene name was given based on the hits. Genes that encode for proteins shorter than 100aa or had no BLASTp hit were ignored from the analysis. The gene order of PAR genes was visualized using Bioconductor Gviz v1.48.0[39].

Testis conserved and cattle specific gene expression

The RNA-seq data quality was checked by FASTQC v0.11.4[40]. The Trim_Galore[41] (v0.4.2) and AdapterRemoval v2.2.1[42] were used for trimming (--quality 10 --clip_R1 10 --clip_R2 10) and adapter removal, respectively. The cleaned reads were aligned to their respective reference genomes using HISAT2 v2.1.0[43] (Supplementary Table 29). The bam files were sorted with SAMtools v1.10. We used Stringtie v2.2.3[44] to generate transcripts per million (TPM) and fragments per kilobase million (FPKM) normalised values and featureCounts v.1.5.2[45] to generate a counts matrix. Genes with a counts per million (CPM) value > 0.5 in at least three samples were retained for further analysis, except for chimpanzee, which we allowed for (CPM) > 0.5 in at least two samples. The OrthoFinder (v2.5.5) was used to identify the orthogroups among cattle, humans, chimpanzees, gorillas, and Bornean orangutans, and the conserved testis-expressed genes were reported. We also searched for tissue expression result of certain cattle-specific genes, such as *Bda20* and *Obp*, using the CattleGTEx database[46].

10x coverage is insufficient to study centromere variation

Mapped BAM files from the 20 Wagyu samples were converted to BED files with bedtools bamtobed. We then intersected this BED file with the centromere and non-centromere coordinates of UOA_Wagyu_1_Y to determine the region (centromere or non-centromere) that each read mapped to. To make the centromere and non-centromere regions comparable within a given chromosome due to the difference in length, we used the following equation:

$$Normalised\_score = total\_mapq / max\_mapq$$

Where total_mapq was the sum of all mapQ scores for reads within the region and max_mapq is the number of reads mapped to that region multiplied by 60 (the maximum mapQ score).

Given UOA_Wagyu_1_Y represents the most complete cattle genome to date, we sought to assess whether centromeric variation could be reliably investigated using ONT reads at 10X coverage. We assessed this using the mapping quality (mapQ) from all mapped reads from the 20 Wagyu samples, all reads ≥ 10 kb in length and all reads ≥ 20 kb in length. In all cases, mapping quality was insufficient for reliable downstream analysis of centromeric variation (Supplementary Fig. 22).

MCG method

To verify that our MCG method selected a more genetically diverse set of 20 individuals versus 50 randomly chosen sets of the same sample size, we counted the number of unique variants in these sets. The number of unique variants referred to AB and BB genotypes at each chromosome position. The genotype data for this analysis came from Versa50K genotyping SNP arrays.

Long-read sequencing of 20 Wagyu samples

Models used for base calling and methylated CpG calling were:

```
dna_r10.4.1_e8.2_400bps_sup@v4.2.0
dna_r10.4.1_e8.2_400bps_sup@v4.2.0_5mCG_5hmCG@v2
```

The full command was:

```
./dorado-0.6.0-linux-x64/bin/dorado basecaller -v -b 0 --min-
qscore 9 ./dorado-0.6.0-linux-
x64/models/dna_r10.4.1_e8.2_400bps_SUP@v4.2.0 --modified-bases-
models ./dorado-0.6.0-linux-
x64/models/dna_r10.4.1_e8.2_400bpsSUP@v4.2.0_5mCG_5hmCG@v2 $pod5
> $output_bam
```

Detection of high-confidence SVs

Dorado uses minimap2[11] as the aligner with the '-x' flag set to 'map-ont', and is able to align the unmapped BAM files without first converting to FASTQ and retains the 'MM' and 'ML' methylation tags from the unmapped bam file in the alignments. The command was:

```
dorado aligner -t 16 --mm2-preset “map-ont” $reference
$unmapped_bam
```

Clair3 was used with the r1041_e82_400bps_sup_v420 model and platform parameter set to 'ont'. We used default parameters for Sniffles2 (v.2.3.2)[47]. CuteSV (v2.1.0)[48] was used with the following parameters: "--max_cluster_bias_INS 100 --max_cluster_bias_DEL 100 --diff_ratio_merging_INS 0.3 --diff_ratio_merging_DEL 0.3 --min_support 5 --min_size 50 –genotype". DYSGU[49] (v1.6.2) was used with "—-mode nanopore" and SVIM[50] (v2.0.0) was used with "--min_mapq 20 --min_sv_size 50 --minimum_depth 5 --insertion_sequences" parameters. The full commands are also available at https://github.com/DaviesCentreInformatics/LR-variantCaller.

SURVIVOR (v1.0.7)[51] was then used to merge SVs and keep those with two or more callers supporting the SV using the parameters '50 2 1 0 0 50'.

Pangenome graph construction and SV extraction

We constructed a pangenome graph using 13 assemblies, Tuli (TUL), Wagyu (WAG), Brahman (BRA), Angus (ANG), Original Braunvieh (OBR), Brown Swiss (BSW), Simmental (SIM), Charolais (CHA), Piedmontese (PIE), Ankole (ANK), N'dama (NDA), Highland (HIL) and ARS-UCD2.0 (HER). For each autosome, we constructed a graph that contained 13 assemblies; for the X chromosome, only 10 of the 13 assemblies contained an X chromosome thus the number of assemblies in this graph was 10. Similarly, the Y chromosome graph contained only five assemblies. The 13 assemblies were split into their individual chromosomes with 'faSplit byname $assembly.fa assembly_chr_dir/'. Chromosomes were renamed following the PanSN prefix naming pattern (https://github.com/pangenome/PanSN-spec). For example, chromosome 1 from UOA_Wagyu_1_Y was renamed from '1' to 'WAG#1#1', chromosome 2 was renamed to 'WAG#1#2' and so on. We then created one multifasta file per chromosome, where each assembly had its respective chromosome as a record within the file. We then constructed pangenome graphs for each chromosome using PGGB[52] (v. e8dd3fd). An example of the command is below:

```
pggb -i $fasta -o HER_"$chrom"_graph -t 8 -p 90 -s 5k -V
'HER#1#$chrom'
```

We then extracted SVs for each assembly from the graph using ODGI (v0.8.6)[53] with the following command:

```
odgi untangle -t 8 -P -i chromosome_"$num".graph.og \
        -r HER#1#"$num" \
        | sed '1d' \
        | cut -f 4,5,6 \
        | sort \
        | uniq \
        | sort -k 2n > cattle.HER_"$num".PAVs.bed &&
odgi pav -M -t 8 -i chromosome"$num".graph.og \
        -b cattle.HER_"$num".PAVs.bed \
   > cattle.HER_"$num".PAVs.mat
```

As UOA_Wagyu_1_Y was used as the backbone of the graph, we removed the "WAG" column from the PAV matrix files and filtered PAVs to only retain those with at least 90% identity in at least one other assembly. PAVs that were only found in UOA_Wagyu_1_Y were also extracted as Wagyu-specific PAVs. These PAVs files were then reformatted as BED files and used as input for hotspotter (https://github.com/daewoooo/primatR). We repeated this for the X chromosome except without Tuli, Ankole and Angus as these assemblies lacked an X chromosome and for the Y chromosome with only Tuli, Angus and Ankole.

Supplementary Figures

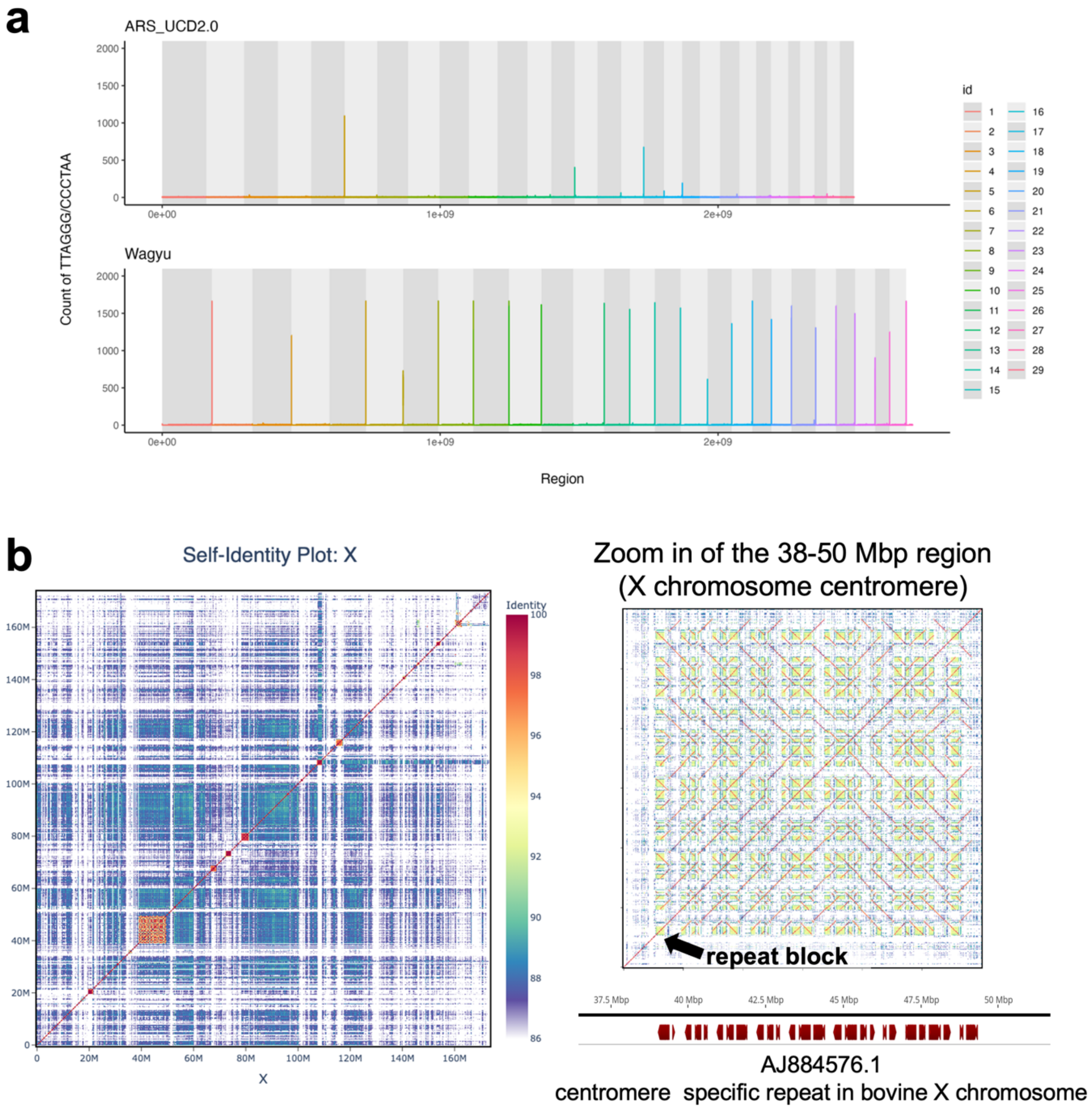

Supplementary Fig. 1. Telomere and X chromosome visualisation. a, A bedgraph of the telomeric repeat signals at a 10-kb window of UOA_Wagyu_1 and ARS-UCD_2.0 using tidk0.2.0. The alternating gray shades delineate the boundaries of each chromosome. **b,** Moddotplot visualization of X chromosome. Black arrow indicates an example of a low sequence similarity repeat block. The X centromere specific repeat (red box) shows alignment to this region with sequence identity >90%, confirming the location the X centromere.

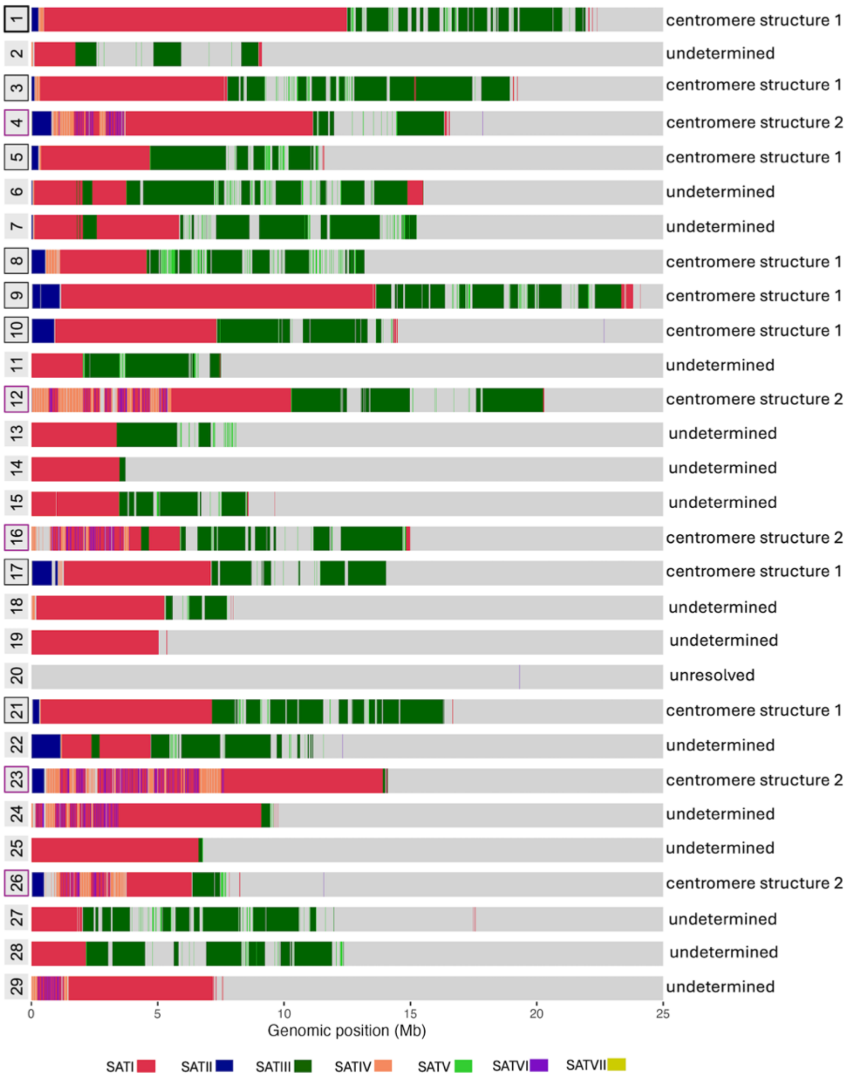

Supplementary Fig. 2. Centromere structure of all autosomes. Each color in the bar represents the seven satellite repeats. Chromosomes boxed in black represents structure 1 that is characterized by patterns of SATII, SATIV, SATI and interspersed SATIII and SATV. Chromosomes boxed in violet represents structure 2 that is characterized by patterns of SATII, SATIV, interspersed SATI and SATVI and tandem repeats of SATI. These are for all autosomes with complete and incomplete centromeres. Chromosomes boxed in gray remain undetermined due to the absence of SATII, which may or may not have interspersed SATI and SATVI. Their centromere structure category is also indicated. The centromere in BTA20 is not resolved.

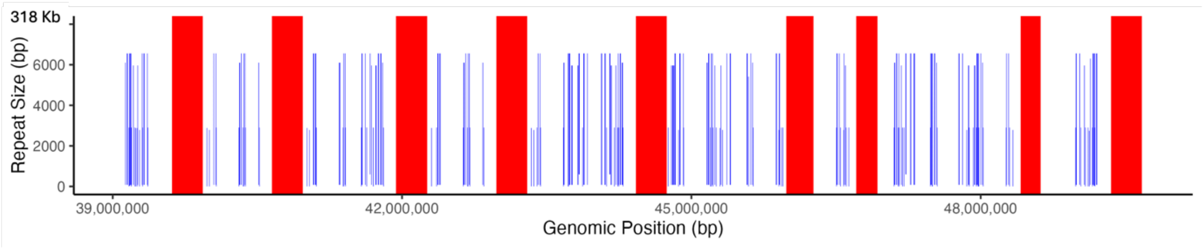

Supplementary Fig. 3. Location of the X centromere specific repeats. The blue line shows the 2,898 bp (XCTR1) and 6,558 bp (XCTR2) repeats, whereas the red bars show the positions of the XCTR3 in the X chromosome.

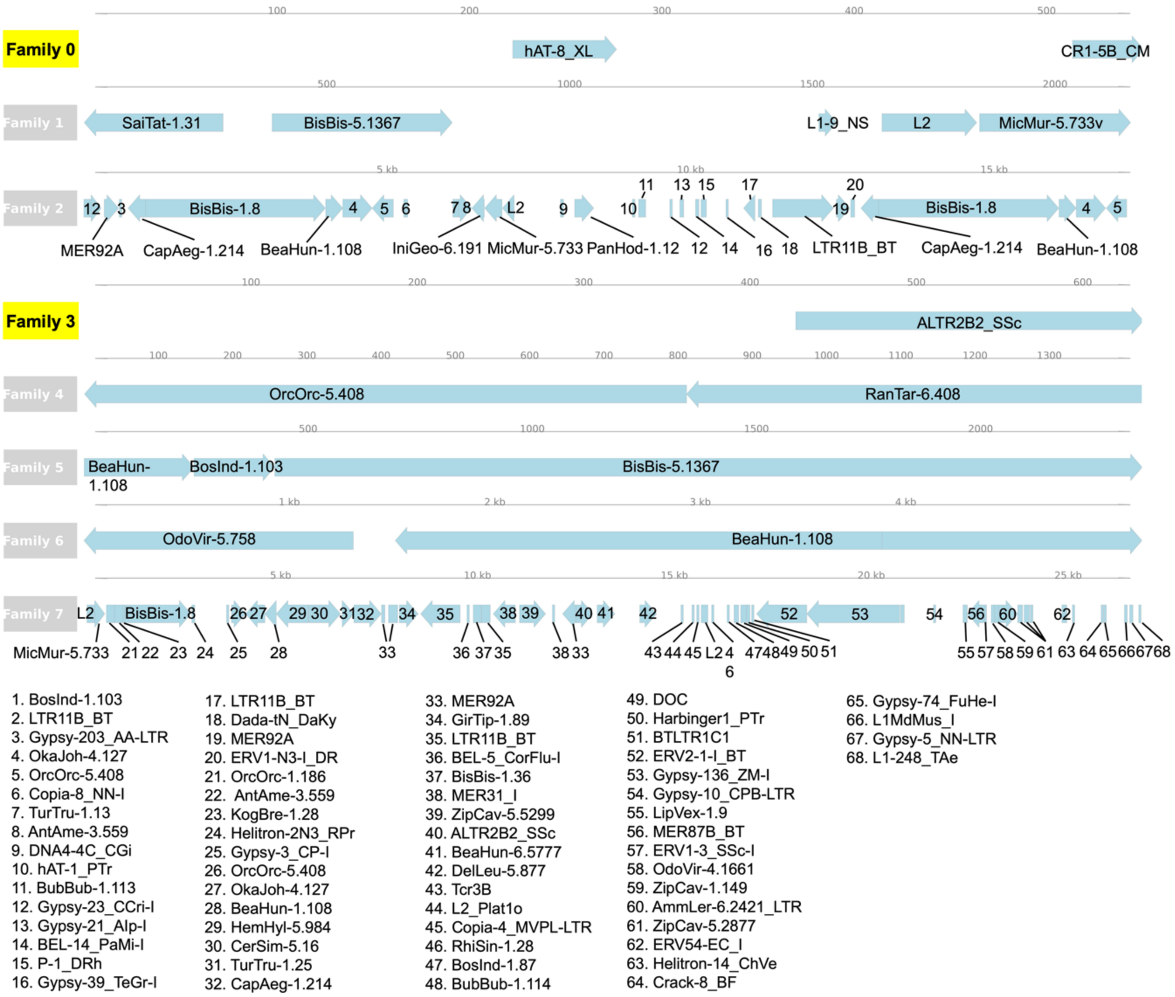

Supplementary Fig. 4. Transposable elements in the X chromosome. Location of transposable elements (TEs) in the comprehensive ab initio repeat pipeline (CARP) consensus sequences of the X centromere region, analyzed under 99% sequence similarity and a minimum length of 400 bp. This figure illustrates the eight consensus sequences identified within the 12 Mb centromere region. The yellow boxes indicate the consensus family specific to the X centromere, blue boxes indicate regions covered by transposable elements based on RepBase data, with arrows representing the direction of the matched transposable element sequences. Arrows pointing to the right indicate a 5' to 3' direction, while those pointing left indicate the reverse. Three consensus sequences are chimeric, with two fully covered by TE fragments aligned in the same direction. These chimeric sequences, originating from diverse transposons, display unique structural characteristics.

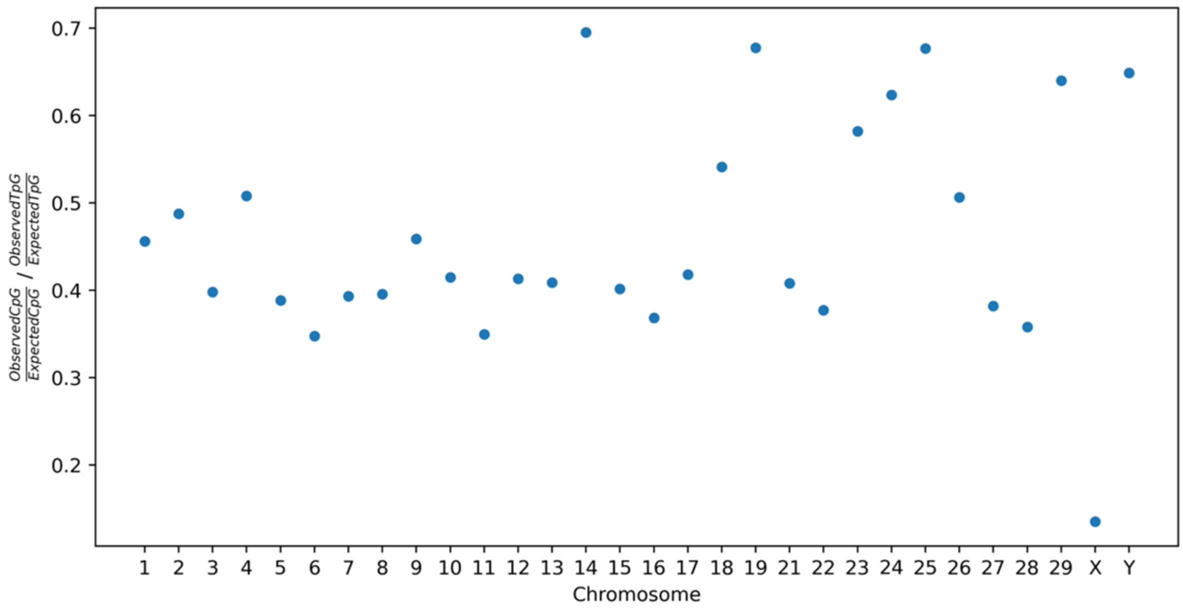

Supplementary Fig. 5. Ratio of ratios for observed (Obs) and expected (Exp) CpGs compared to observed and expected TpGs. Scatterplot showing the ratios of (Obs_CpG/Exp_CpG) / (Obs_TpG/Exp_TpG) for each centromere. BTA20 is missing due to not being able to confidently identify the centromere. The Y-axis denotes the ratio and the X-axis denotes the chromosomes.

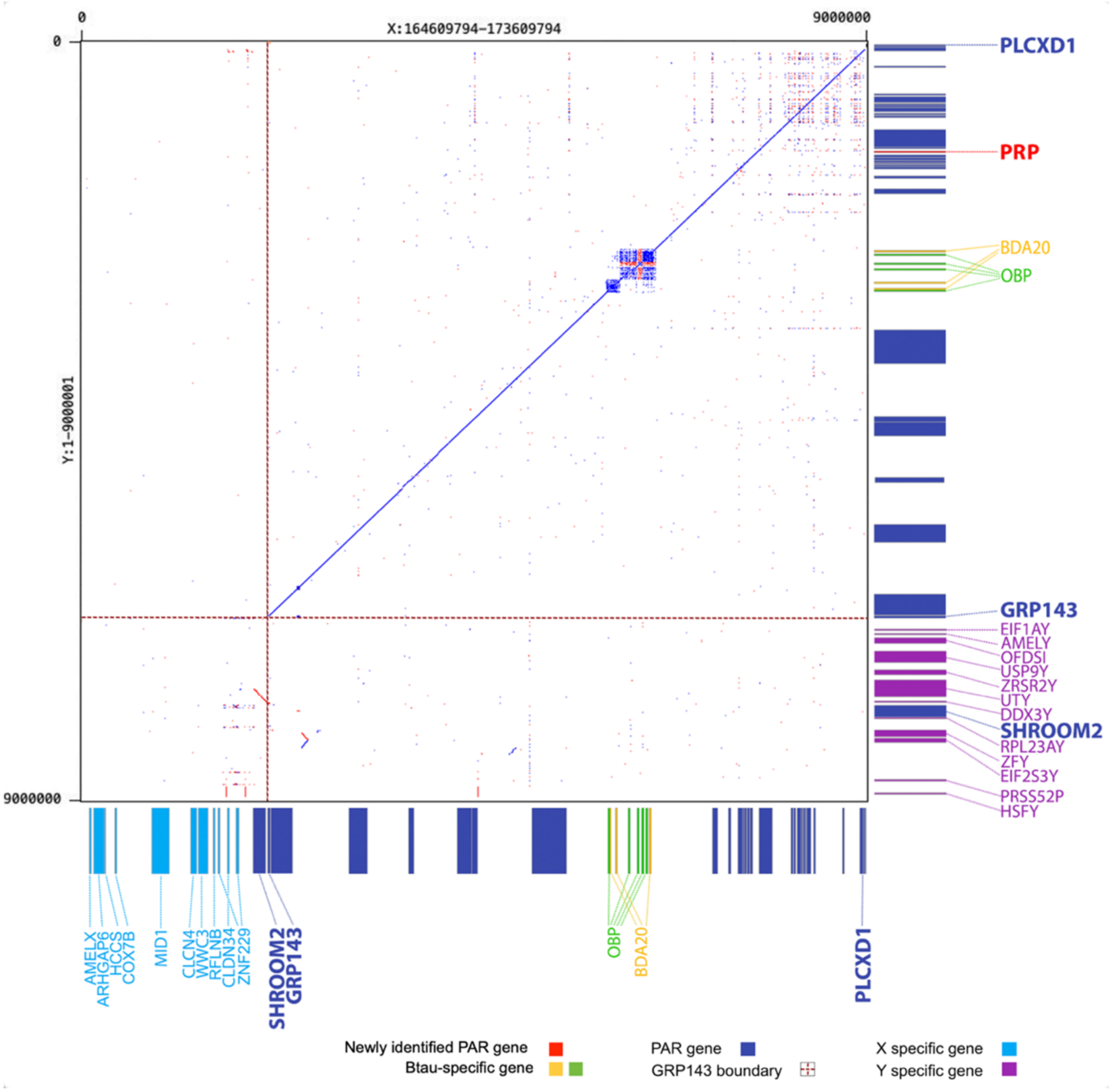

Supplementary Fig. 6. Comparison of the Wagyu X and Y cattle chromosome pseudoautosomal regions (PARs) and adjacent sequence. The blue dots are forward alignment, and red dots are reversed alignment. The x-axis is X chromosome and y-axis is Y chromosome. The cattle PAR on X is between X: 166757707-173598413 (reversed) and Y: 20023-6819277 according to the alignment. The X and Y chromosome PAR genes (dark blue) share the same gene content and gene order. Gene families *OBP* and *BDA20* are highlighted in green and yellow. New gene *PRP* identified on Y chromosome is highlighted in red. *SHROOM2* is located distal to the pseudoautosomal boundary (PAB) on the BTAX, which is found inverted on the Y chromosome X-d1 region. Color scale of the heatmap from blue to red indicates increasing sequence similarity.

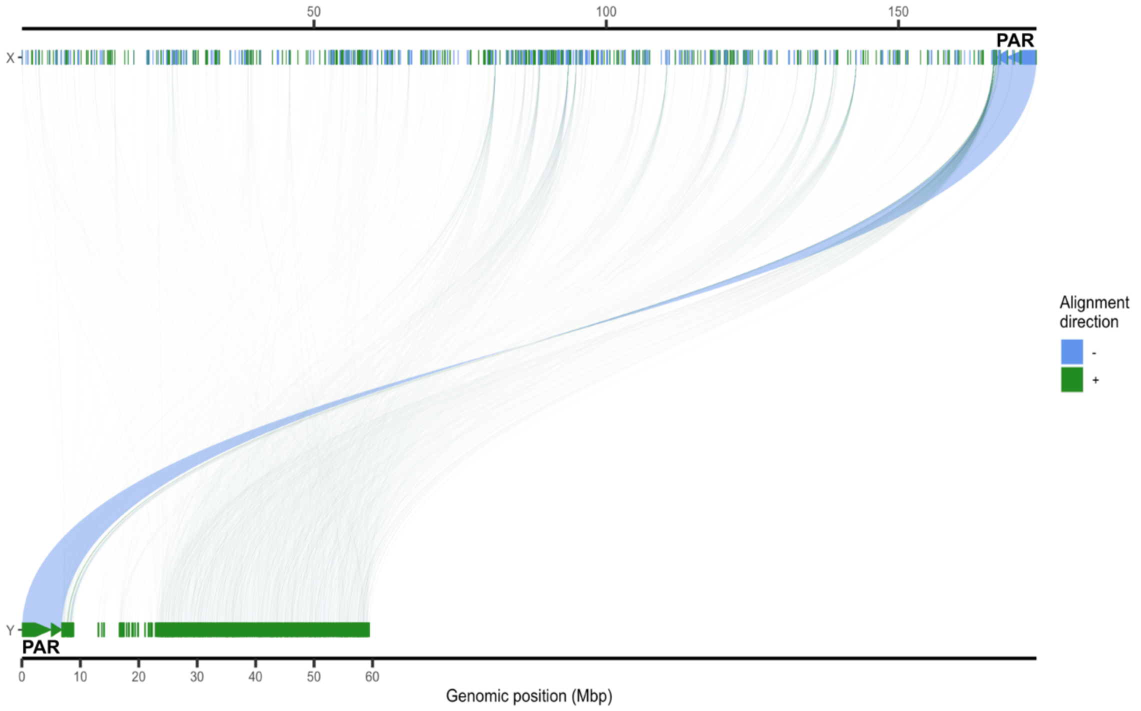

Supplementary Fig. 7. The alignment plot between X and Y chromosomes. The PAR region aligns at both termini of the X and Y chromosome but at opposite ends (q-arm vs p-arm). Blue color indicates opposite orientation (-) whereas green indicates forward orientation (+).

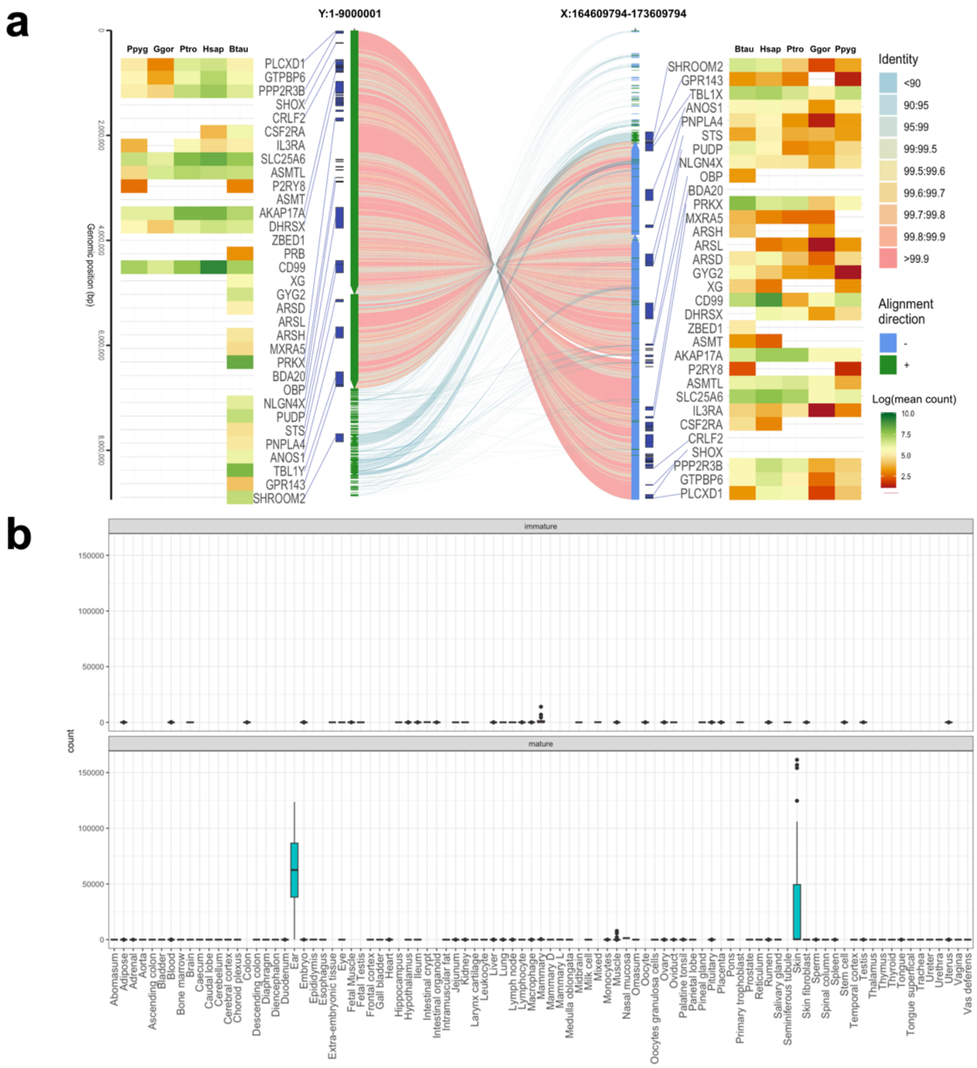

Supplementary Fig. 8. Gene expression. a, Testes-expressed genes in sex chromosome for PAR regions across species. The genes are listed according to the order of cattle genes. Btau is *Bos taurus*, Hsap is *Homo sapiens*, Ptro is *Pan troglodytes*, Ggor is *Gorilla gorilla* and Ppyg is *Pongo pygmaeus*. **b,** The expression of *BDA20* (ENSBTAG00000016820) in cattle tissues grouped for mature and immature cattle. We grouped the mature cattle equal to or older than one year old. There were 3107 samples of 91 types of tissues on the boxplot. Color scale of the heatmap from red to green indicates increasing mean count on a logarithmic scale.

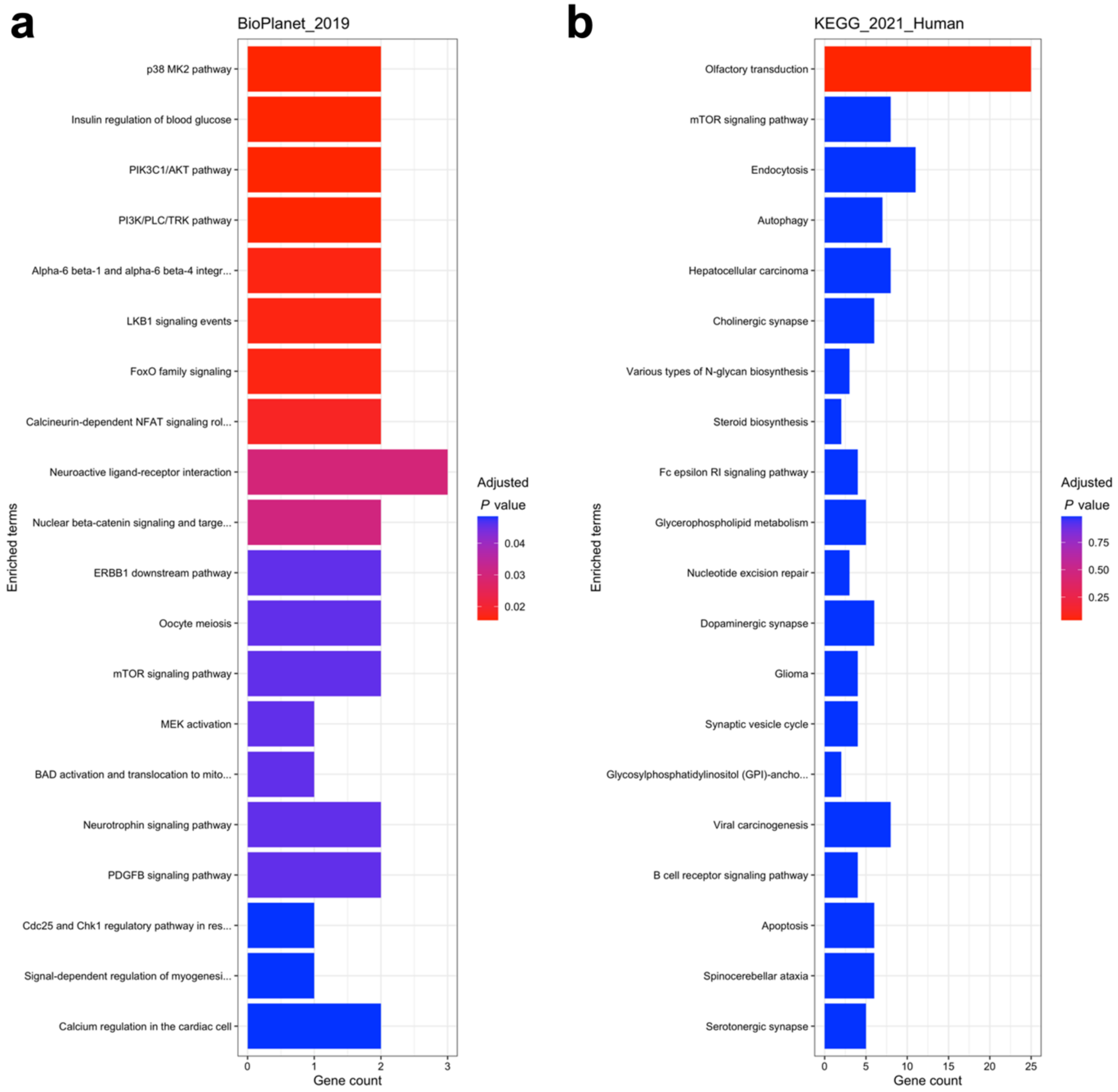

Supplementary Fig. 9. Pathway enrichment analysis. a, Pathway enrichment for genes within the X centromere. **b,** Pathway enrichment of genes that overlap with Wagyu-specific hotspots. Enriched terms identified among the 509 protein-coding genes that overlap with Wagyu-specific structural variant hotspots. Adjusted P value of Olfactory transduction = 0.04. Color scale from red to blue indicates decreasing adjusted P value.

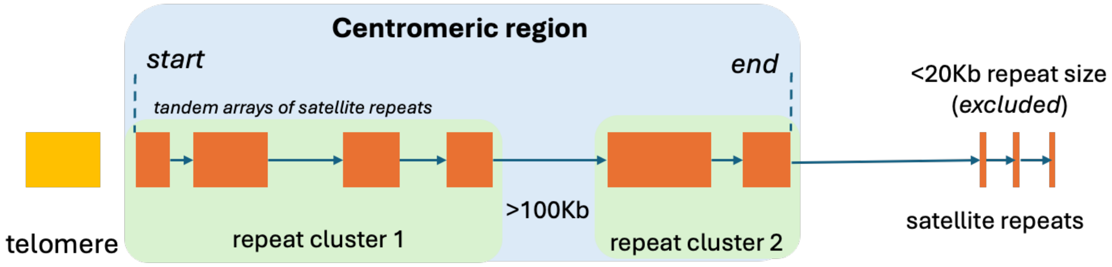

Supplementary Fig. 10. Centromeric repeat clusters in autosomes. The figure illustrates the method in identifying the centromeric region (blue box) through bovine satellite repeat clusters (green box). Given the acrocentric chromosomes of the cattle, the telomere region (yellow box) is adjacent to the bovine satellite repeats (orange box) which marks the start of the centromeric region. Repeat clusters were identified as consecutive satellite repeats within 100Kb distance. The end boundary was identified as the last instance of the satellite repeat within the repeat cluster. Consecutive satellite repeats within the 100 kb but were <20Kb in total size were excluded and not classified as a repeat cluster.

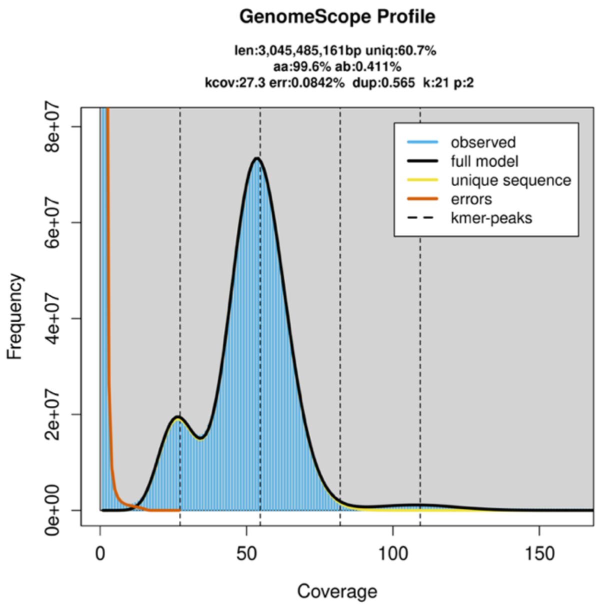

Supplementary Fig. 11. **GenomeScope Profile**. K-mer analysis profile of the UOA_Wagyu_1 exhibiting two peaks indicating a heterozygous genome using GenomeScope2. The first peak (first dashed line) represents heterozygous with 25x coverage (single copy) and the second peak (second dashed line), represents homozygous k-mers at 50x coverage (duplicated copies).

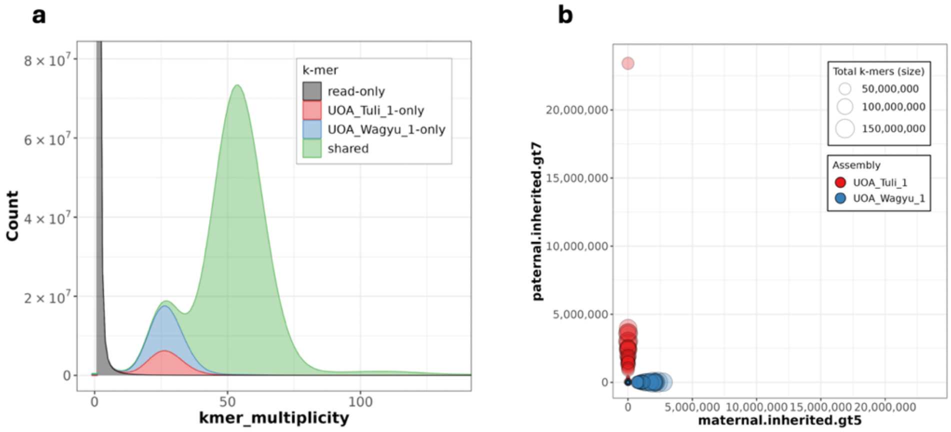

Supplementary Fig. 12. **Genome quality assessment plot of the Wagyu and Tuli genome assemblies generated using Merqury**. (a) The k-mer spectra plots of the haplotype-resolved genome assemblies displaying the k-mer distribution of the F1 read set to its corresponding haplotypes. The red graph represents Tuli assembly-specific k-mers, the blue graph shows Wagyu-specific k-mers, and the green graph depicts k-mers shared between the two breeds. (b) The hapmer blob plot showing the effective phasing of the assemblies. The size of the bubble represents the size of the total k-mers.

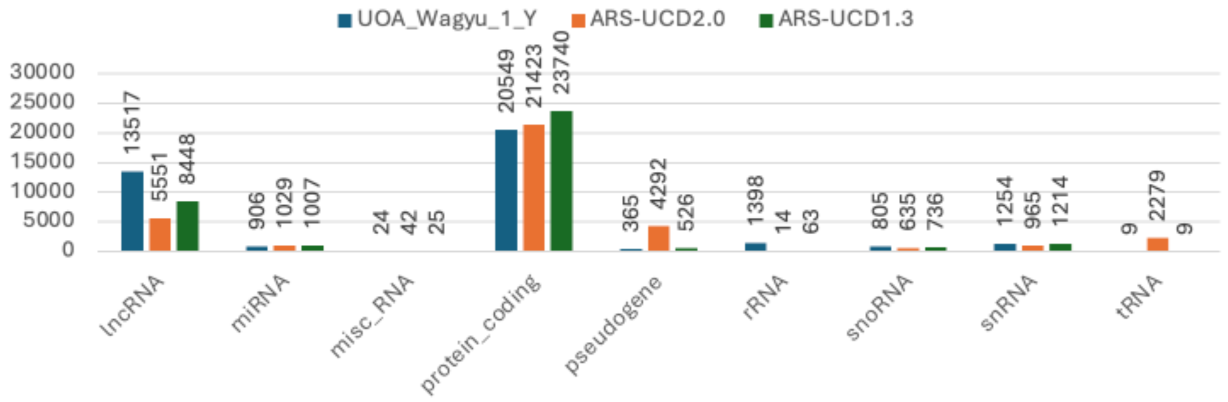

Supplementary Fig. 13. Gene annotation comparison of three assemblies. Bar plot showing the different frequencies of annotations across UOA_Wagyu_1_Y (blue), ARS-UCD2.0 (orange) and ARS-UCD1.3 (green). The X-axis denotes the different annotations, and the Y-axis denotes the count. The data value is above each bar.

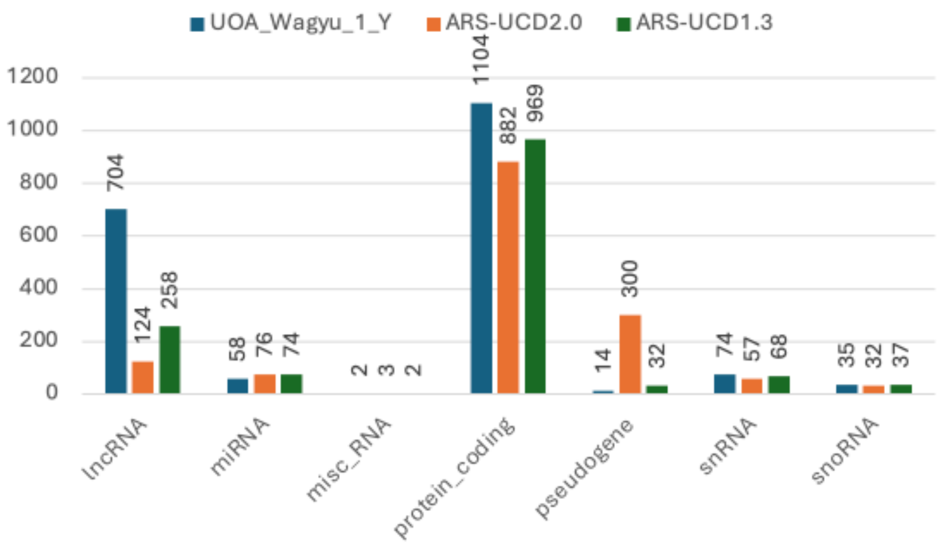

Supplementary Fig. 14. Gene annotation comparison of three assemblies focusing on the X chromosome. Bar plot showing the different frequencies of annotations across UOA_Wagyu_1_Y (blue), ARS-UCD2.0 (orange) and ARS-UCD1.3 (green). The X-axis denotes the different annotations, and the Y-axis denotes the count. The data value is above each bar.

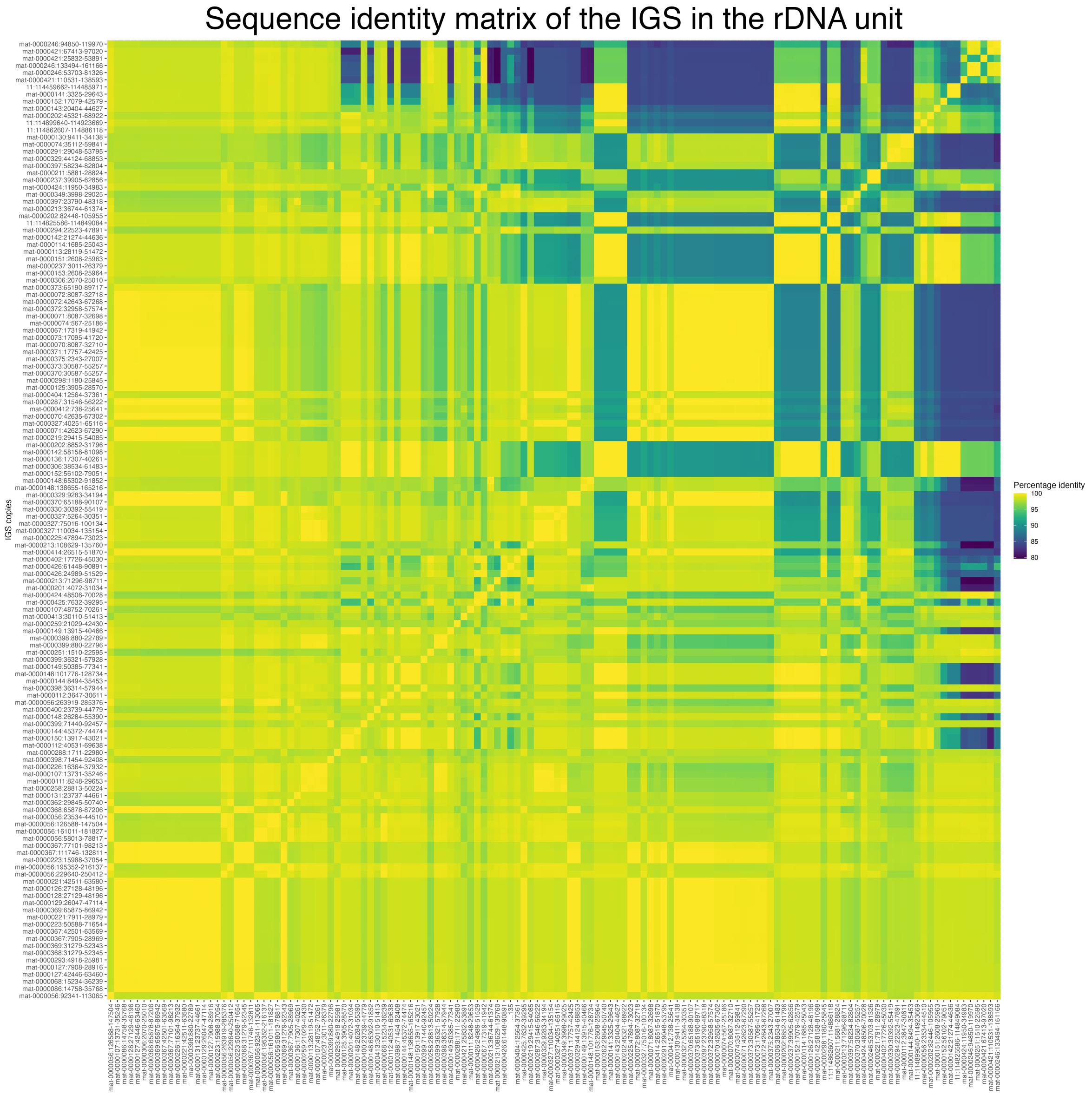

Supplementary Fig. 15. **Sequence identity matrix of the intergenic spacer (IGS) within the 35 kb rDNA**. The heatmap matrix displays visualisation of the pairwise similarity of each IGS unit. Color scale from blue to yellow indicates increasing sequence similarity.

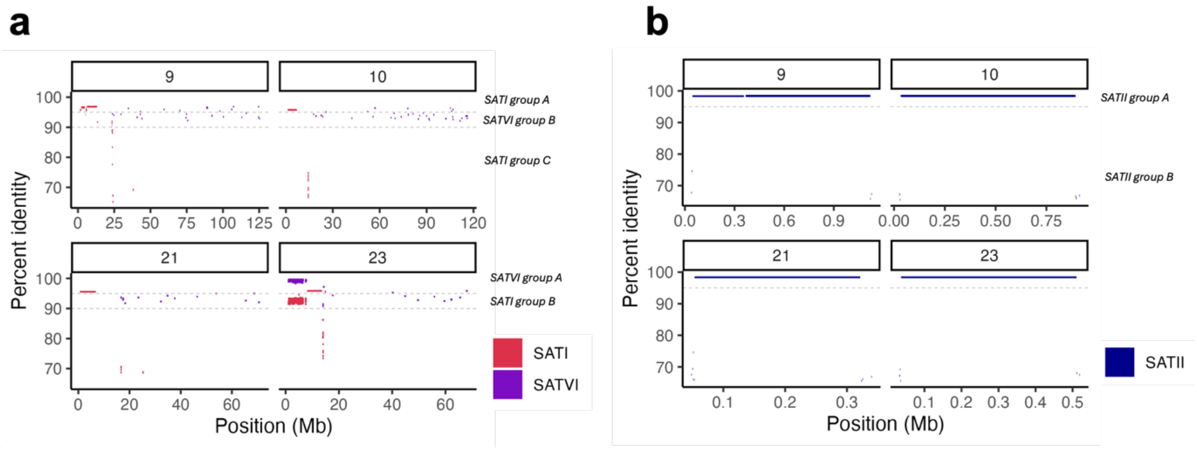

Supplementary Fig. 16. Different groups of the SATI, SATII and SATVI repeat satellites based on their percent identity in our T2T chromosomes. The plot shows the different groups of satellite repeats. a, SATI into three as GroupA (≥95%), GroupB (<95% & >90%), GroupC (≤90%) and SATVI into two as GroupA (≥98%) and GroupB (<98%). The horizontal line is a guide for the 90% and 95% sequence identity. b, two group group of SATII with GroupA (≥98%) and GroupB (<98%). The different groups of the satellite repeats are associated with specific locations in the centromere, and across the genome for SATVI group B.

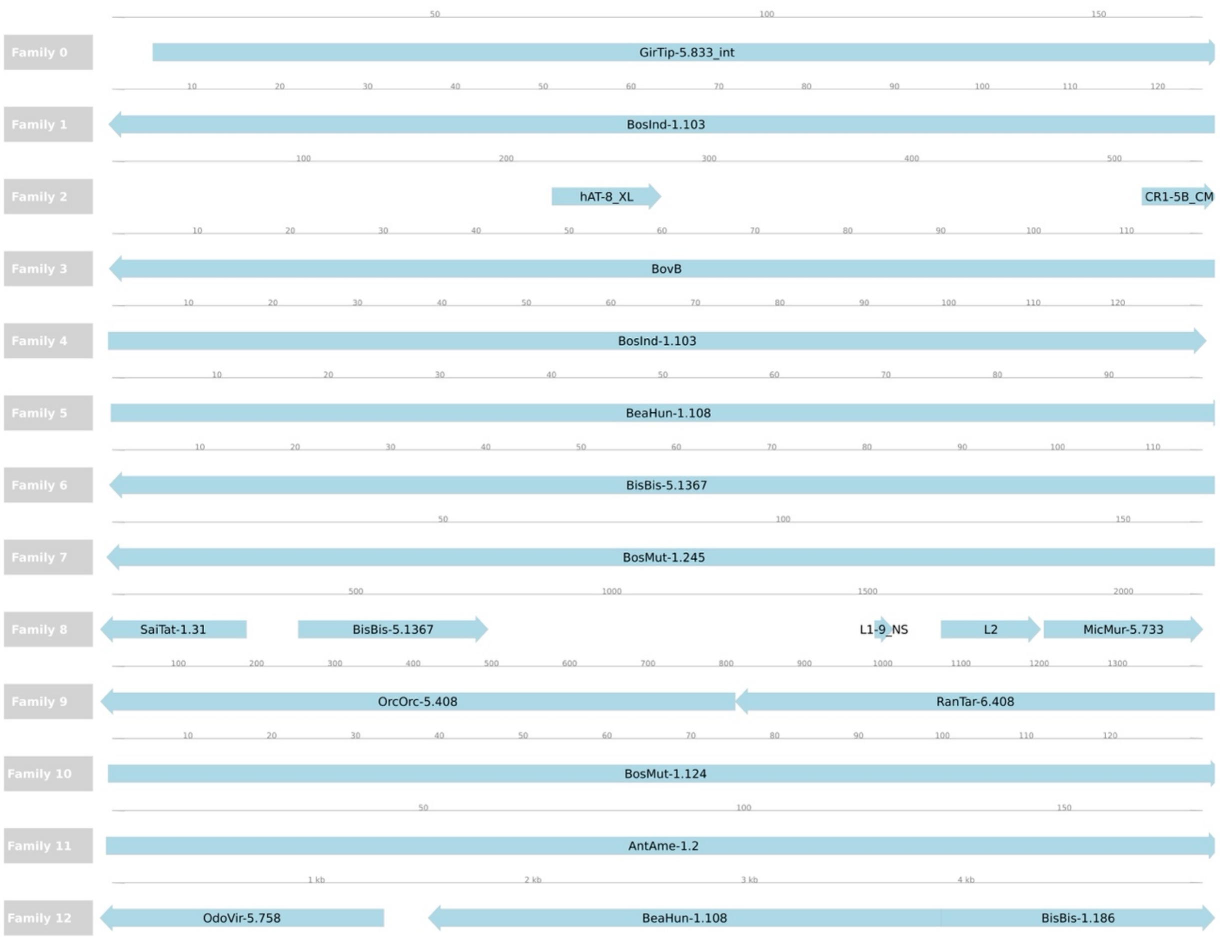

Supplementary Fig. 17. **Location of transposable elements in the CARP** consensus sequences of the X centromere region, analyzed under 99% sequence similarity and a minimum length of 100 bp.

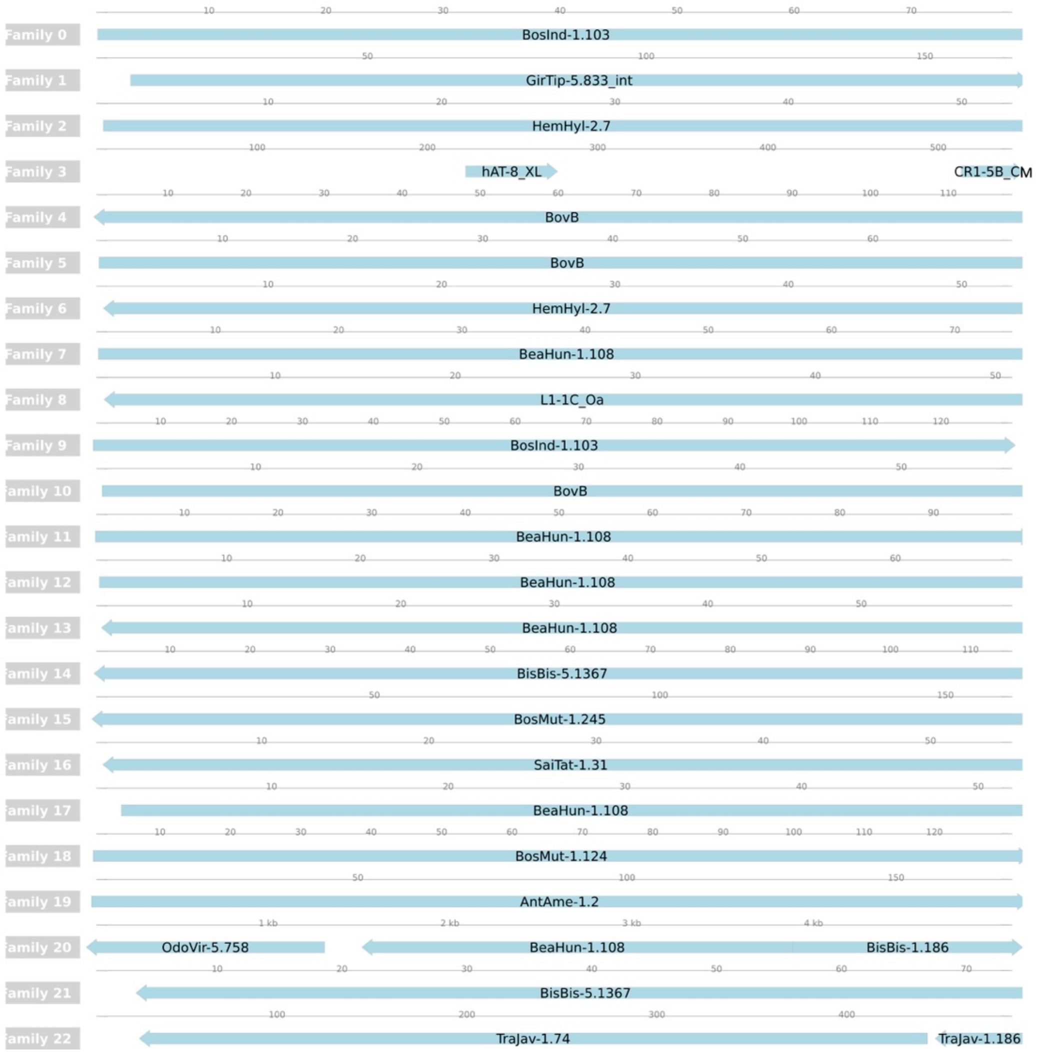

Supplementary Fig. 18. Location of transposable elements in the CARP consensus sequences of the X centromere region, analyzed under 99% sequence similarity and a minimum length of 50 bp.

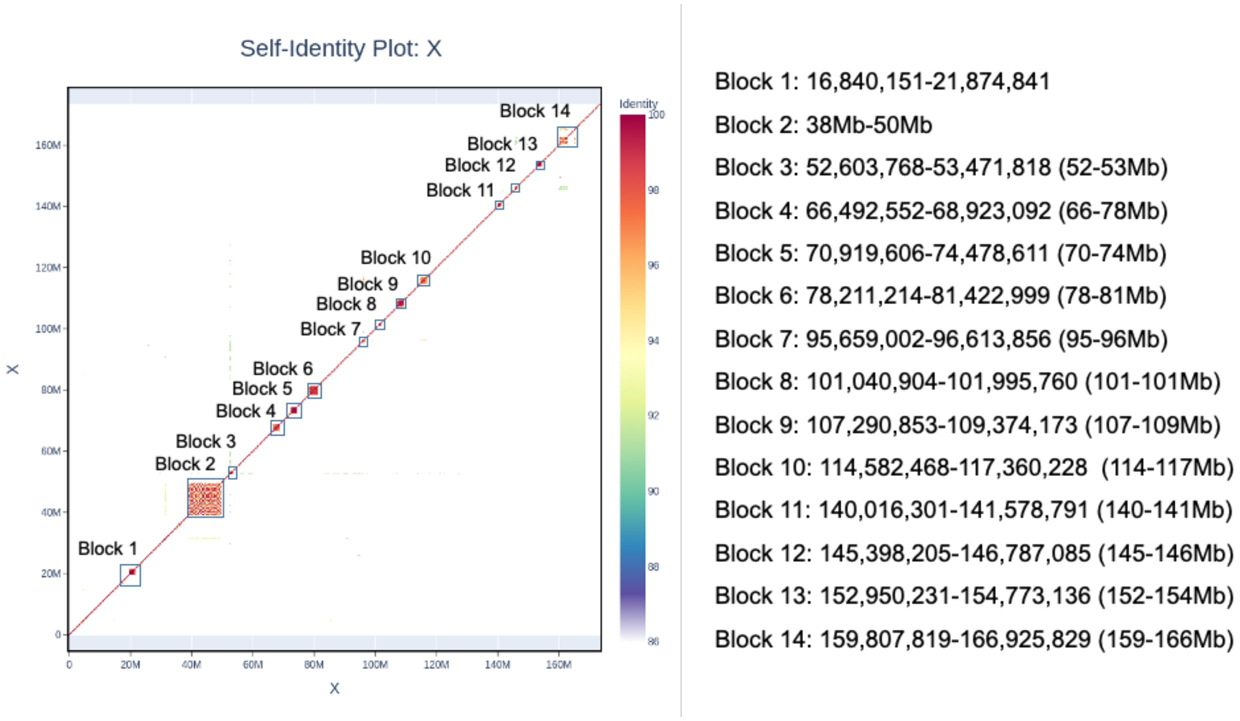

Supplementary Fig. 19. **X chromosome self-alignment plot generated using ModDotPlot**. The plot displays 14 distinct repeat blocks of varying lengths within the X chromosome region, filtered to show only sequences with greater than 90% similarity. The color gradient on the right represents sequence identity, with red indicating near-perfect identity and cooler colors reflecting lower sequence similarity. Block 2, corresponding to the centromere region of X chromosome, shows a high density of repeats, particularly short tandem repeats, as observed in the self-alignment analysis. The other labeled blocks (Blocks 1–14) indicate regions of significant repeat sequence variation across the chromosome.

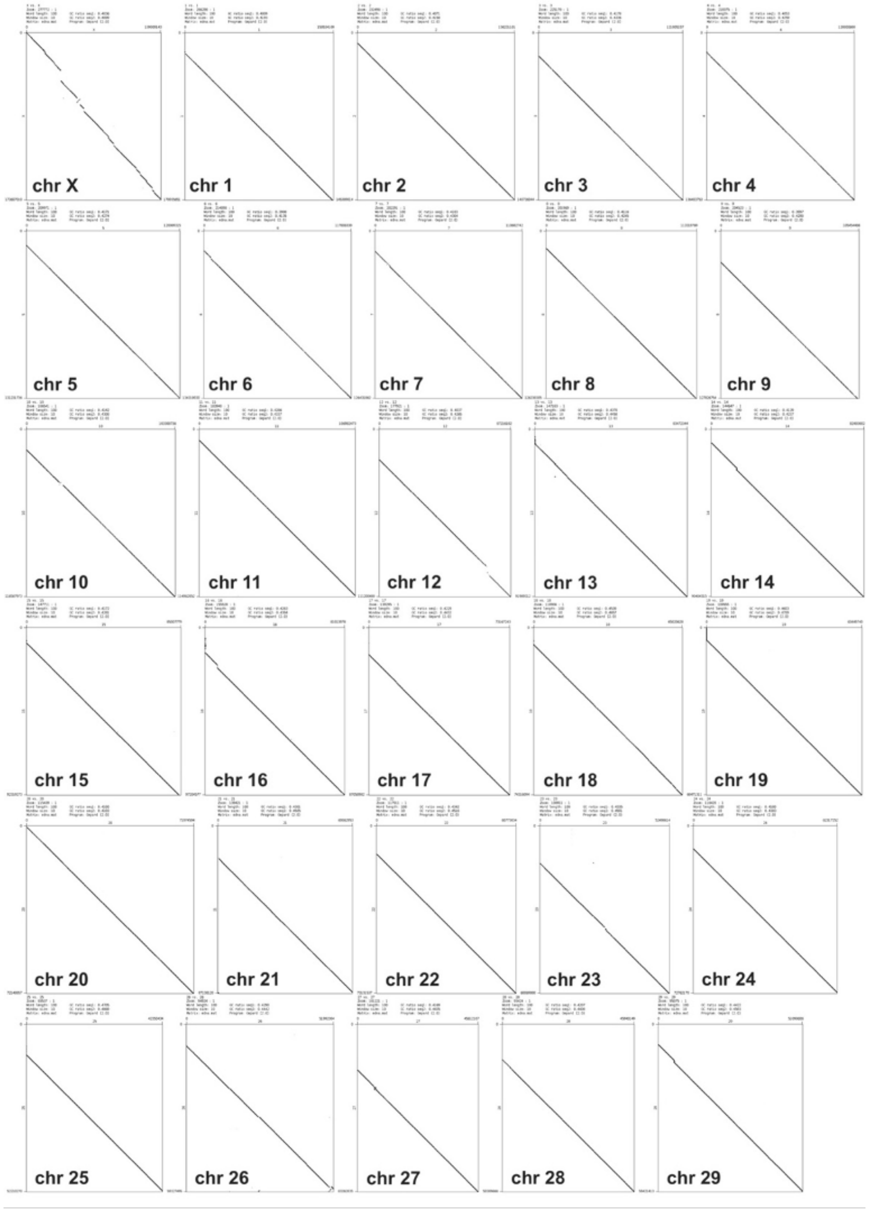

Supplementary Fig. 20. Chromosome homology with ARS-UCD2.0. Dotplot alignment comparing the UOA_Wagyu_1 (y-axis) to the ARS-UCD2.0 (x-axis) for each of the chromosomes using Gepard2.1. The dotplot reveals sequence alignments and correct orientation between chromosomes, with UOA_Wagyu_1 showing additional sequences at the beginning of several of the chromosomes, corresponding to the newly resolved centromeric regions.

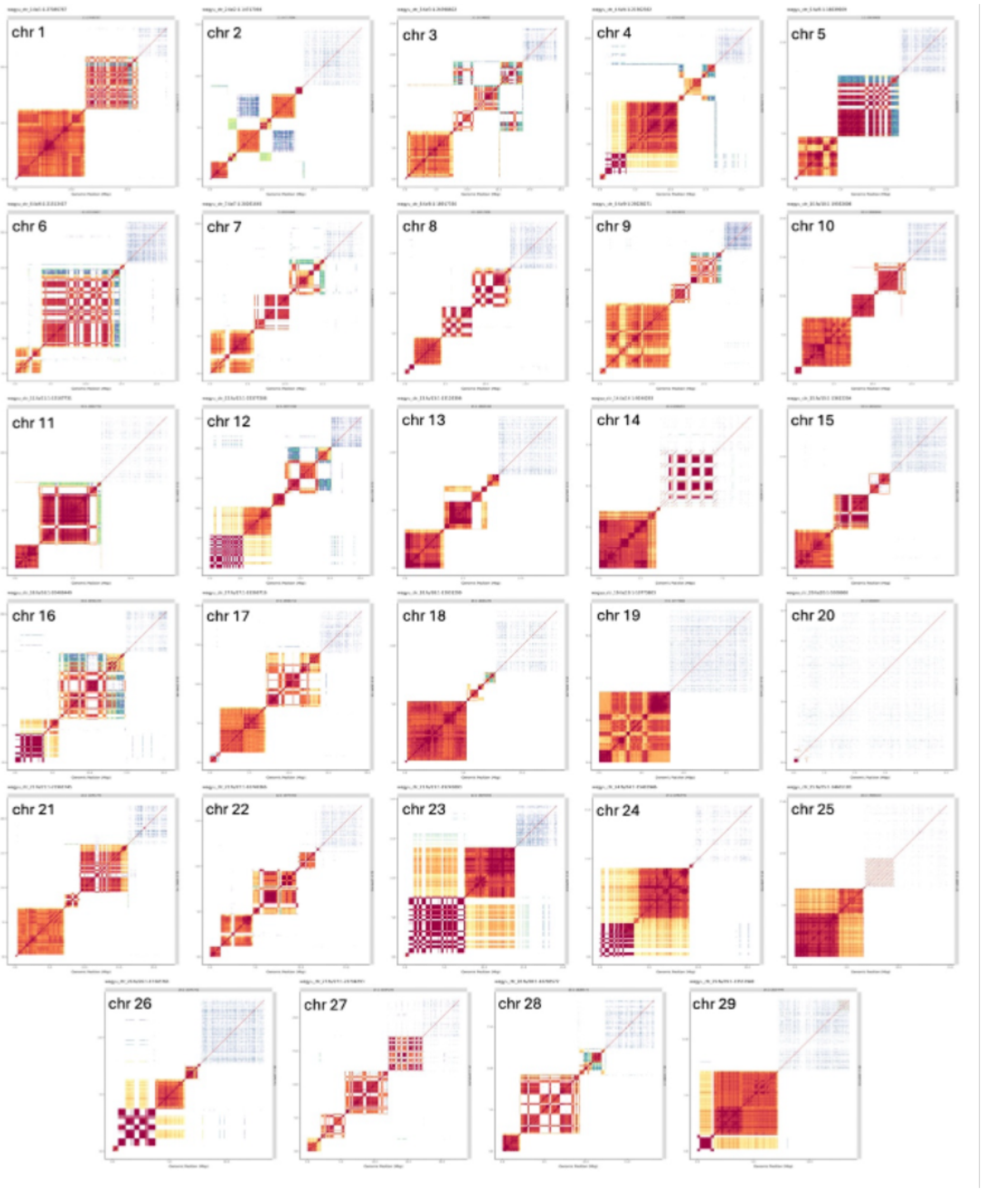

Supplementary Fig. 21. Centromeric region self-alignment dotplot. Self-alignment of the centromeric region with 5Mb upstream sequence using Moddotplot. The centromeric region were not properly assembled for chromosome 20, and hence the lack of highly identical repeat sequences (red blocks) compared to other chromosomes. Color scale from green to red indicates increasing sequence similarity.

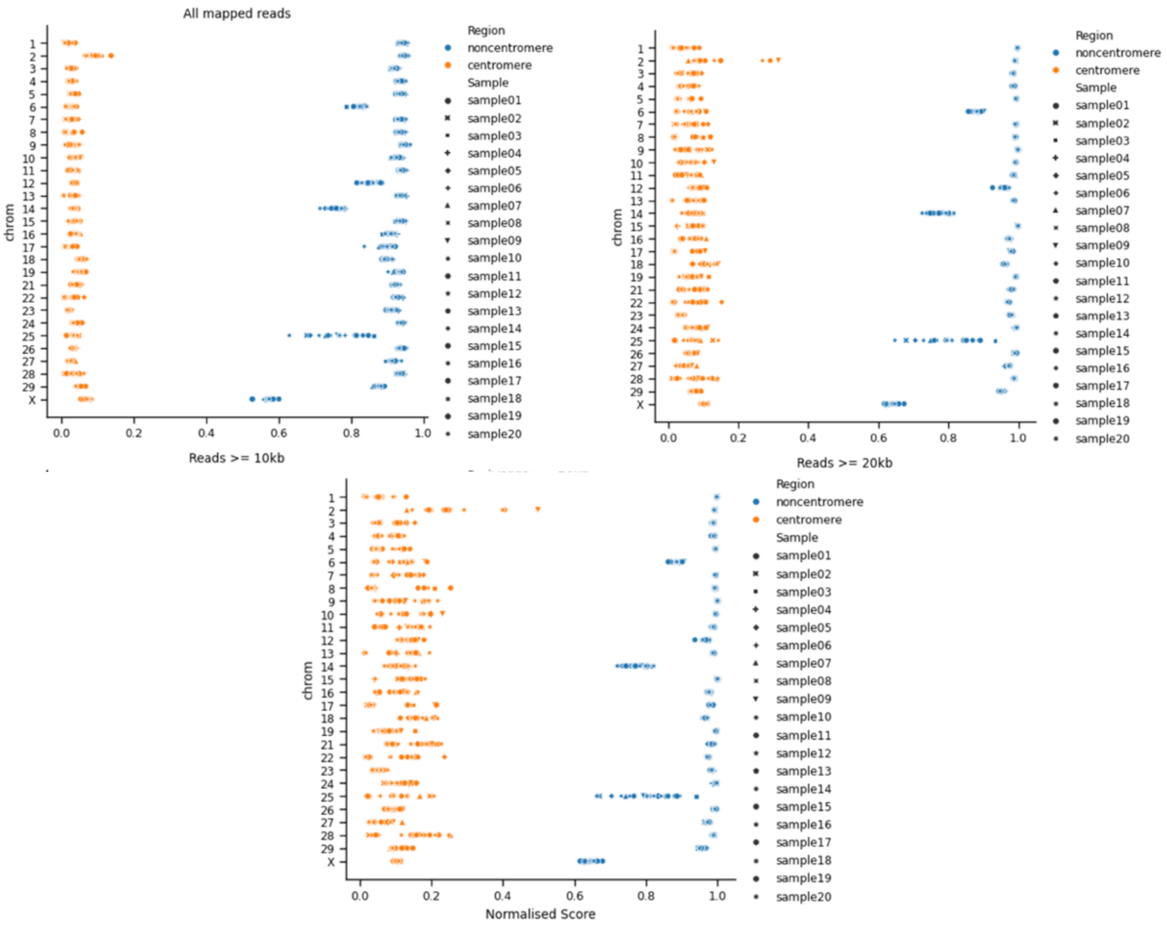

Supplementary Fig. 22. Comparison of read mapping qualities (mapQ) of reads mapping to the centromere and non-centromere at three different read lengths. top left) all mapped reads available in each sample. Colours represent whether the read aligned to a centomere or non-centromere region, with symbols representing different samples. The X-axis denotes the normalised mapQ score for the region and sample and the Y-axis denotes the chromosomes. Top right) same as top left) but only considering reads ≥ 10 kb. bottom) same as top right) but only considering reads ≥ 20 kb.

Supplementary Table

Supplementary Table 1. GC content of the UOA_Wagyu_1 and ARS-UCD2.0 chromosomes and the UOA_Wagyu_1 centromeric region.

Chromosome	UOA_Wagyu_1	ARS-UCD2.0	UOA_Wagyu_1 centromere
1	42.00%	40.00%	55.30%
2	41.50%	40.50%	53.90%
3	43.50%	42.00%	53.10%
4	42.50%	40.50%	57.50%
5	42.50%	41.50%	53.30%
6	41.50%	40.00%	51.30%
7	43.00%	42.00%	51.80%
8	42.50%	41.00%	52.90%
9	43.00%	40.00%	55.60%
10	43.00%	41.50%	54.80%
11	43.50%	43.00%	51.20%
12	42.50%	40.50%	52.70%
13	44.50%	43.50%	53.90%
14	42.50%	41.50%	57.50%
15	43.00%	41.50%	53.30%
16	44.00%	42.50%	51.30%
17	44.50%	42.50%	54.60%
18	46.50%	45.50%	55.90%
19	47.00%	46.00%	57.70%
20	41.00%	41.00%	-
21	45.00%	43.00%	53.30%
22	45.00%	43.50%	54.00%
23	46.00%	43.50%	56.10%
24	44.00%	42.00%	55.70%
25	48.50%	47.00%	55.50%
26	44.50%	43.00%	53.80%
27	44.50%	41.50%	51.20%
28	44.00%	42.00%	50.60%
29	46.00%	44.00%	56.00%
X	41.00%	40.50%	42.50%
Y		45.00%	
Mean	43.80%	42.30%	53.70%

Supplementary Table 2. The GC content of the different bovine satellite repeats in the centromeric region

**Satellite repeat**	**Total alignment**	**GC content**	**GC content (%)**
SATI	151842691	90763034	58.60%
SATII	7425001	4932902	55.80%
SATIII	114179016	54483510	49.30%
SATIV	11258652	5820057	51.80%
SATV	10507422	5601132	53.50%
SATVI	5968365	2638428	44.20%
SATVII	16924	9319	60.20%

Supplementary Table 3. Gene annotations of the different cattle assemblies from chromosomes 1 to X and Y. Yellow highlighted rows denote rows that were common to Ensembl and NCBI annotations. These rows were used to generate Supplementary Figure 13.

	ARS-UCD2.0	UOA_Wagyu_1_Y	ARS-UCD1.3
**biotype**	**Count**	**Count**	**Count**
antisense_RNA	1	-	-
C_region	18	-	-
lncRNA	5551	13517	8448
miRNA	1029	906	1007
misc_RNA	42	24	25
ncRNA	28	-	-
other	3	-	-
protein_coding	21423	20549	23740
pseudogene	4292	365	526
RNase_MRP_RNA	1	-	-
rRNA	14	1398	63
snoRNA	635	805	736
snRNA	965	1254	1214
SRP_RNA	1	-	-
telomerase_RNA	1	-	-
transcribed_pseudogene	59	1	
tRNA	2279	9	9
V_segment	104	-	-
IG_C_gene	-	4	13
IG_D_gene	-	3	-
IG_J_gene	-	2	-
IG_V_gene	-	39	4
TR_C_gene	-	8	3
TR_J_gene	-	24	20
TR_V_gene	-	203	54
Y_RNA	-	15	15
processed_pseudogene	-	26	64
ribozyme	-	6	6
scaRNA	-	29	30
vault_RNA	-	8	8

Supplementary Table 4. Gene annotations of the different cattle assemblies in X chromosome. Yellow highlighted rows denote rows that were common to Ensembl and NCBI annotations. These rows were used to generate Supplementary Figure 14.

biotype	UOA_Wagyu_1_Y	ARS-UCD2.0	ARS-UCD1.3
lncRNA	704	124	258
miRNA	58	76	74
misc_RNA	2	3	2
processed_pseudogene	5	-	-
protein_coding	1104	882	969
pseudogene	14	300	32
rRNA	2	-	2
ribozyme	1	-	1
scaRNA	1	-	1
snRNA	74	57	68
snoRNA	35	32	37
vault_RNA	2	-	2
ncRNA	-	1	-
misc_RNA	-	3	-
tRNA	-	111	-
transcribed_pseudogene		3	

Supplementary Table 5. The coordinates and status of the 5S rDNA region found at the p-arm of the chromosomes.

Chromosome	Coordinates (Mb)		Repeat block size (Mb)	Status
	Start	End		
14	4.3	7	2.7	complete
22	11.8	12.3	0.51	with gaps
27	12.2	17.4	5.22	complete
28	15.2	15.3	0.09	with gaps

Supplementary Table 6. The coordinates and level of the highest CENP-A peak found in the X chromosome. Normalised CENP-A level is computed by dividing CENP-A level with the size of the region.

Region			CENP-A level		Tandem repeat content	
Start	End	Size	Broad	Normalised	Size (bp)	Copies
26805318	26825765	20448	6260760	306.18	18	1140
110806249	111293932	487683	1119302	28.97	53	
141051169	141072658	21490	2831786	131.77	24	892
140489889	140515436	25548	1574938	61.65	24	1036
135795417	135804788	9372	1216205	129.77	no tandem repeat	
140877301	140899223	21923	1153459	52.61	24	643

Supplementary Table 7. CARP annotation of consensus sequences with 100 bp minimum length and 99% sequence identity.

Family ID	Classification condition	Annotation details
family000000	Fully	GirTip-5.833_int (96)
family000001	Fully	BosInd-1.103 (100)
family000002	PartialAnnotation	hAT-8_XL (10); CR1-5B_CM (7)
family000003	Fully	KAF7251931.1 (100)
family000004	Fully	BosInd-1.103 (98)
family000005	Fully	BeaHun-1.108 (100)
family000006	Fully	XP_028869771.1 (100)
family000007	Fully	BosMut-1.245 (100)
family000008	PartialAnnotation	BisBis-5.1367 (17); MicMur-5.733 (14); SaiTat-1.31 (13); L2 (9)
family000009	Chimeric	OrcOrc-5.408 (57); RanTar-6.408 (43)
family000010	Fully	BosMut-1.124 (100)
family000011	Fully	AntAme-1.2 (100)
family000012	Chimeric	BeaHun-1.108 (46); OdoVir-5.758 (25); BisBis-1.186 (25)

Supplementary Table 8. CARP annotation of consensus sequences with 50 bp minimum length and 99% sequence identity.

**Family ID**	**Classification condition**	**Annotation details**
Family 0	Fully	BosInd-1.103 (100)
Family 1	Fully	GirTip-5.833_int (96)
Family 2	Fully	HemHyl-2.7 (100)
Family 3	PartialAnnotation	hAT-8_XL (10); CR1-5B_CM (7)
Family 4	Fully	KAF7251931.1 (100)
Family 5	Fully	BovB (100)
Family 6	Fully	HemHyl-2.7 (100)
Family 7	Fully	BeaHun-1.108 (100)
Family 8	Fully	L1-1C_Oa (100)
Family 9	Fully	BosInd-1.103 (98)
Family 10	Fully	BovB (100)
Family 11	Fully	BeaHun-1.108 (100)
Family 12	Fully	BeaHun-1.108 (100)
Family 13	Fully	BeaHun-1.108 (100)
Family 14	Fully	XP_028869771.1 (100)
Family 15	Fully	BosMut-1.245 (100)
Family 16	Fully	SaiTat-1.31 (100)
Family 17	Fully	BeaHun-1.108 (98)
Family 18	Fully	BosMut-1.124 (100)
Family 19	Fully	AntAme-1.2 (100)
Family 20	Chimeric	BeaHun-1.108 (46); OdoVir-5.758 (25); BisBis-1.186 (25)
Family 21	Fully	BisBis-5.1367 (96)
Family 22	Chimeric	TraJav-1.74 (84); TraJav-1.186 (10)

Supplementary Table 9. Repeat blocks found chromosome X through visualization in moddotplot. An asterisk (*) indicates the centromeric region.

Block	Start (bp)	End (bp)	Length (bp)	Length in Mb
1	16,840,151	21,874,841	5,034,690	5.03
2*	38,000,000	50,000,000	12,000,000	12
3	52,603,768	53,471,818	868,050	0.87
4	66,492,552	68,923,092	2,430,540	2.43
5	70,919,606	74,478,611	3,559,005	3.56
6	78,211,214	81,422,999	3,211,785	3.21
7	95,659,002	96,613,856	954,854	0.95
8	101,040,904	101,995,760	954,856	0.95
9	107,290,853	109,374,173	2,083,320	2.08
10	114,582,468	117,360,228	2,777,760	2.78
11	140,016,301	141,578,791	1,562,490	1.56
12	145,398,205	146,787,085	1,388,880	1.39
13	152,950,231	154,773,136	1,822,905	1.82
14	159,807,819	166,925,829	7,118,010	7.12

Supplementary References

1. Rautiainen, M. *et al.* Telomere-to-telomere assembly of diploid chromosomes with Verkko. *Nature Biotechnology* **41**, 1474-+ (2023).
2. Cheng, H.Y., Concepcion, G.T., Feng, X.W., Zhang, H.W. & Li, H. Haplotype-resolved de novo assembly using phased assembly graphs with hifiasm. *Nature Methods* **18**, 170-+ (2021).
3. Stanojević, D., Lin, D., Florez de Sessions, P. & Šikić, M. Telomere-to-telomere phased genome assembly using error-corrected Simplex nanopore reads. *bioRxiv*, 2024.05.18.594796 (2024).
4. Rosen, B.D. *et al.* De novo assembly of the cattle reference genome with single-molecule sequencing. *Gigascience* **9**(2020).
5. Uliano-Silva, M. *et al.* MitoHiFi: a python pipeline for mitochondrial genome assembly from PacBio high fidelity reads. *BMC Bioinformatics* **24**, 288 (2023).
6. Schmidt, T.T. *et al.* High resolution long-read telomere sequencing reveals dynamic mechanisms in aging and cancer. *Nature Communications* **15**, 5149 (2024).
7. Band, M.R. *et al.* An ordered comparative map of the cattle and human genomes. *Genome Res* **10**, 1359-68 (2000).
8. Pauciullo, A. *et al.* Isolation and physical localization of new chromosome-specific centromeric repeats in farm animals. *Veterinarni medicina* **51**, 224-231 (2006).
9. Perucatti, A. *et al.* Advanced comparative cytogenetic analysis of X chromosomes in river buffalo, cattle, sheep, and human. *Chromosome Res* **20**, 413-25 (2012).
10. Liu, R. *et al.* New insights into mammalian sex chromosome structure and evolution using high-quality sequences from bovine X and Y chromosomes. *BMC Genomics* **20**, 1000 (2019).
11. Li, H. Minimap2: pairwise alignment for nucleotide sequences. *Bioinformatics* **34**, 3094-3100 (2018).
12. Bickhart, D.M. CombineFasta. Vol. 2022 (2020).
13. Krumsiek, J., Arnold, R. & Rattei, T. Gepard: a rapid and sensitive tool for creating dotplots on genome scale. *Bioinformatics* **23**, 1026-8 (2007).
14. Danecek, P. *et al.* Twelve years of SAMtools and BCFtools. *Gigascience* **10**(2021).
15. Poplin, R. *et al.* A universal SNP and small-indel variant caller using deep neural networks. *Nat Biotechnol* **36**, 983-987 (2018).
16. Rhie, A. *et al.* Towards complete and error-free genome assemblies of all vertebrate species. *Nature* **592**, 737-746 (2021).
17. Gurevich, A., Saveliev, V., Vyahhi, N. & Tesler, G. QUAST: quality assessment tool for genome assemblies. *Bioinformatics* **29**, 1072-5 (2013).
18. Rhie, A., Walenz, B.P., Koren, S. & Phillippy, A.M. Merqury: reference-free quality, completeness, and phasing assessment for genome assemblies. *Genome Biol* **21**, 245 (2020).
19. Li, H. seqtk. (2018).
20. Tang, D. *et al.* Cloning and sequencing of the complicated rDNA gene family of Bos taurus. *Czech Journal of Animal Science* **51**(2006).
21. Hori, Y.A.-O., Shimamoto, A. & Kobayashi, T.A.-O. The human ribosomal DNA array is composed of highly homogenized tandem clusters. (2021).
22. Shen, W., Le, S., Li, Y. & Hu, F. SeqKit: A Cross-Platform and Ultrafast Toolkit for FASTA/Q File Manipulation. *PLOS ONE* **11**, e0163962 (2016).

23. Katoh, K. & Standley, D.M. MAFFT Multiple Sequence Alignment Software Version 7: Improvements in Performance and Usability. *Molecular Biology and Evolution* **30**, 772-780 (2013).
24. Finn, R.D. *et al.* HMMER web server: 2015 update. *Nucleic Acids Research* **43**, W30-W38 (2015).
25. Wick, R.R., Schultz, M.B., Zobel, J. & Holt, K.E. Bandage: interactive visualization of de novo genome assemblies. *Bioinformatics* **31**, 3350-2 (2015).
26. Olagunju, T.A. *et al.* Telomere-to-telomere assemblies of cattle and sheep Y-chromosomes uncover divergent structure and gene content. *Nat Commun* **15**, 8277 (2024).
27. Langmead, B., Trapnell, C., Pop, M. & Salzberg, S.L. Ultrafast and memory-efficient alignment of short DNA sequences to the human genome. *Genome Biol* **10**, R25 (2009).
28. Meers, M.P., Tenenbaum, D. & Henikoff, S. Peak calling by Sparse Enrichment Analysis for CUT&RUN chromatin profiling. *Epigenetics Chromatin* **12**, 42 (2019).
29. Marcais, G. & Kingsford, C. A fast, lock-free approach for efficient parallel counting of occurrences of k-mers. *Bioinformatics* **27**, 764-70 (2011).
30. Smit, A.H., R; Green, P. . RepeatMasker Open-4.0. (2013-2015).
31. Thorvaldsdóttir, H., Robinson, J.T. & Mesirov, J.P. Integrative Genomics Viewer (IGV): high-performance genomics data visualization and exploration. *Briefings in Bioinformatics* **14**, 178-192 (2013).
32. Kokot, M., Długosz, M. & Deorowicz, S. KMC 3: counting and manipulating k-mer statistics. *Bioinformatics* **33**, 2759-2761 (2017).
33. Zhang, Y., Chu, J., Cheng, H. & Li, H. De novo reconstruction of satellite repeat units from sequence data. *Genome Res* **33**, 1994-2001 (2023).
34. Benson, G. Tandem repeats finder: a program to analyze DNA sequences. *Nucleic Acids Research* **27**, 573-580 (1999).
35. Sweeten, A.P., Schatz, M.C. & Phillippy, A.M. ModDotPlot-rapid and interactive visualization of tandem repeats. *Bioinformatics* **40**(2024).
36. Quinlan, A.R. & Hall, I.M. BEDTools: a flexible suite of utilities for comparing genomic features.
37. Harris, R. (2007).
38. Kielbasa, S.M., Wan, R., Sato, K., Horton, P. & Frith, M.C. Adaptive seeds tame genomic sequence comparison. *Genome Res* **21**, 487-93 (2011).
39. Hahne, F. & Ivanek, R. Visualizing Genomic Data Using Gviz and Bioconductor. (2016).
40. Andrews, S. FastQC: A Quality Control Tool for High Throughput Sequence Data. *https://www.bioinformatics.babraham.ac.uk/projects/fastqc/* (2010).
41. Krueger, F. TrimGalore. (https://github.com/FelixKrueger/TrimGalore, 2021).
42. Schubert, M., Lindgreen, S. & Orlando, L. AdapterRemoval v2: rapid adapter trimming, identification, and read merging. *BMC Res Notes* **9**, 88 (2016).
43. Kim, D., Paggi, J.M., Park, C., Bennett, C. & Salzberg, S.L. Graph-based genome alignment and genotyping with HISAT2 and HISAT-genotype. *Nat Biotechnol* **37**, 907-915 (2019).
44. Shumate, A., Wong, B., Pertea, G. & Pertea, M. Improved transcriptome assembly using a hybrid of long and short reads with StringTie. *PLoS Comput Biol* **18**, e1009730 (2022).
45. Liao, Y., Smyth, G.K. & Shi, W. featureCounts: an efficient general purpose program for assigning sequence reads to genomic features. *Bioinformatics* **30**, 923-30 (2014).

46. Fang, L. *et al.* Comprehensive analyses of 723 transcriptomes enhance genetic and biological interpretations for complex traits in cattle. *Genome Res* **30**, 790-801 (2020).
47. Smolka, M. *et al.* Detection of mosaic and population-level structural variants with Sniffles2. *Nature Biotechnology* (2024).
48. Jiang, T. *et al.* Long-read-based human genomic structural variation detection with cuteSV. *Genome Biol* **21**, 189 (2020).
49. Cleal, K. & Baird, D.M. Dysgu: efficient structural variant calling using short or long reads. *Nucleic Acids Res* **50**, e53 (2022).
50. Heller, D. & Vingron, M. SVIM: structural variant identification using mapped long reads. *Bioinformatics* **35**, 2907-2915 (2019).
51. Jeffares, D.C. *et al.* Transient structural variations have strong effects on quantitative traits and reproductive isolation in fission yeast. *Nat Commun* **8**, 14061 (2017).
52. Garrison, E. *et al.* Building pangenome graphs. *Nat Methods* **21**, 2008-2012 (2024).
53. Guarracino, A., Heumos, S., Nahnsen, S., Prins, P. & Garrison, E. ODGI: understanding pangenome graphs. *Bioinformatics* **38**, 3319-3326 (2022).
