# Supplementary material for: Insights into natural neocentromere evolution from a cattle T2T X chromosome
Source: Nat Commun. 2025 Nov 28;16:10745. doi: 10.1038/s41467-025-65778-w (PMC12663436; doi:10.1038/s41467-025-65778-w)
Supplement: Supplementary file 2 — Description of Additional Supplementary Files [file 41467_2025_65778_MOESM2_ESM.docx]

**Description of Additional Supplementary Files**

| File Name: Supplementary Data 1  Description: Different runs of Verkko and hifiasm with different coverage and sequencing reads |
| --- |
| File Name: Supplementary Data 2  Description: The QV based on Merqury per chromosomes before and after polishing with DeepVariant. |
| File Name: Supplementary Data 3  Description: The number and percentage of repeat families in UOA_Wagyu_1 and ARS-UCD2.0 (autosomes, X, Y, MT and unplaced) |
| File Name: Supplementary Data 4  Description: GFF table and percentage identity and complete copies of the rDNA parts |
| File Name: Supplementary Data 5  Description: Assembly statistics of the UOA_Wagyu_1 per chromosomes |
| File Name: Supplementary Data 6  Description: CENP-A enrichment levels of the bovine satellite repeats called with SEACR within the centromeric region using the broad and max peak |
| File Name: Supplementary Data 7  Description: Summary of bovine satellite repeats for number of copies, total length and quantitative statistics for each autosomes |
| File Name: Supplementary Data 8  Description: Centromere vs Non-centromere methylation values across UOA_Wagyu_1_Y using ONT and PacBio data |
| File Name: Supplementary Data 9  Description: Observed and Expected C and G counts from UOA_Wagyu_1_Y centromere |
| File Name: Supplementary Data 10  Description: Comparison of centromeric C and G counts across seven species |
| File Name: Supplementary Data 11  Description: Protein-coding genes in X-PAR regions for different species |
| File Name: Supplementary Data 12  Description: Protein-coding genes in Y-PAR regions for different species |
| File Name: Supplementary Data 13  Description: Protein-coding genes out of the PAR regions on X and Y chromosomes |
| File Name: Supplementary Data 14  Description: The BlastP results for homologous genes between X and Y chromosomes |
| File Name: Supplementary Data 15  Description: A list of all expressed genes in testis for cattle, human, chimpanzee, gorilla and Borneo orangutan |
| File Name: Supplementary Data 16  Description: The expressed genes in X-PAR for cattle, human, chimpanzee, gorilla, and Borneon orangutan |
| File Name: Supplementary Data 17  Description: The expressed genes in Y-PAR for cattle, human, chimpanzee, gorilla, and Borneon orangutan |
| File Name: Supplementary Data 18  Description: Overview of new annotations in regions previously unseen in ARS-UCD2.0 |
| File Name: Supplementary Data 19  Description: Protein-coding genes found within X centromere |
| File Name: Supplementary Data 20  Description: Protein-coding genes expressed in testis within X centromere for cattle, human, gorilla and Bornean orangutan |
| File Name: Supplementary Data 21  Description: Comparison of ONT mapping rates of 20 Wagyu samples when mapped to ARS-UCD2.0, UOA_Wagyu_1_Y and UOA_Wagyu_1_Y with centromeres masked |
| File Name: Supplementary Data 22  Description: Comparison of short read mapping rate between UOA_Wagyu_1_Y and ARS-UCD2.0 |
| File Name: Supplementary Data 23  Description: Comparison of SNPs identified using ARS-UCD2.0 versus UOA_Wagyu_1_Y |
| File Name: Supplementary Data 24  Description: Frequency and category of different SVs identified in ARS-UCD2.0 and UOA_Wagyu_1_Y |
| File Name: Supplementary Data 25  Description: Frequency and genomic location of SVs identified in ARS-UCD2.0 and UOA_Wagyu_1_Y |
| File Name: Supplementary Data 26  Description: Protein-coding genes that overlap with a Wagyu-specific SV hotspot by at least 50% |
| File Name: Supplementary Data 27  Description: Raw sequencing coverage |
| File Name: Supplementary Data 28  Description: The different bovine satellite repeats and their corresponding names in RepBase and the literatures |
| File Name: Supplementary Data 29  Description: Public RNA-seq data collected for testis expression |
